# SPINDLE: End-to-end learning from EEG/EMG to extrapolate animal sleep scoring across experimental settings, labs and species

**DOI:** 10.1371/journal.pcbi.1006968

**Published:** 2019-04-18

**Authors:** Đorđe Miladinović, Christine Muheim, Stefan Bauer, Andrea Spinnler, Daniela Noain, Mojtaba Bandarabadi, Benjamin Gallusser, Gabriel Krummenacher, Christian Baumann, Antoine Adamantidis, Steven A. Brown, Joachim M. Buhmann

**Affiliations:** 1 Department of Computer Science, ETH Zurich, Zürich, Switzerland; 2 Max Planck Institute for Intelligent Systems, Tübingen, Germany; 3 Chronobiology and Sleep Research Group, University of Zurich, Zürich, Switzerland; 4 Department of Biomedical Sciences, Washington State University, Spokane, Washington, United States of America; 5 Department of Neurology, University Hospital Zurich, Zürich, Switzerland; 6 Department of Neurology, University of Bern, Bern, Switzerland; University of Surrey, UNITED KINGDOM

## Abstract

Understanding sleep and its perturbation by environment, mutation, or medication remains a central problem in biomedical research. Its examination in animal models rests on brain state analysis via classification of electroencephalographic (EEG) signatures. Traditionally, these states are classified by trained human experts by visual inspection of raw EEG recordings, which is a laborious task prone to inter-individual variability. Recently, machine learning approaches have been developed to automate this process, but their generalization capabilities are often insufficient, especially across animals from different experimental studies. To address this challenge, we crafted a convolutional neural network-based architecture to produce domain invariant predictions, and furthermore integrated a hidden Markov model to constrain state dynamics based upon known sleep physiology. Our method, which we named SPINDLE (Sleep Phase Identification with Neural networks for Domain-invariant LEearning) was validated using data of four animal cohorts from three independent sleep labs, and achieved average agreement rates of 99%, 98%, 93%, and 97% with scorings from five human experts from different labs, essentially duplicating human capability. It generalized across different genetic mutants, surgery procedures, recording setups and even different species, far exceeding state-of-the-art solutions that we tested in parallel on this task. Moreover, we show that these scored data can be processed for downstream analyzes identical to those from human-scored data, in particular by demonstrating the ability to detect mutation-induced sleep alteration. We provide to the scientific community free usage of SPINDLE and benchmarking datasets as an online server at https://sleeplearning.ethz.ch. Our aim is to catalyze high-throughput and well-standardized experimental studies in order to improve our understanding of sleep.

## Author summary

Machine learning-based approaches hold great promise to pave the way for high-throughput animal sleep monitoring. With the novel developments of gene-engineering techniques and the proliferation of experimental sleep studies, the need for the automation and cross-lab standardization of sleep scoring becomes more imminent. Traditionally, the classification of electroencephalographic (EEG) signatures is done by trained human experts via visual inspection. Here we present a novel algorithm based upon neural networks to automatically generate accurate and physiologically plausible predictions. Performed experiments demonstrate that the proposed solution offers de facto human level performance, is more accurate than any other approach to date (93-99% accurate compared to multiple trained human scorers), and functions across different genetic mutants, surgery procedures, recording setups and even different species. Moreover, our method was capable of detecting mutation-induced changes in sleeping patterns. To allow for its widespread adaptation, we make our framework freely available through the provision of an online server and an easy to use interface. This community tool will both contribute to the standardization of experimental studies and enhance scientific understanding of sleep.

This is a *PLOS Computational Biology* Methods paper.

## Introduction

The importance of sleep in humans and animals is a widely studied and intriguing topic in medical research [1]. Across all organisms with neurons—from Aplysia “sea slugs” to man—sleep-like states can be identified [2], and in mammalian phyla these basic states share characteristic synchronous neuronal oscillations accompanied by partial or total cessation of motor activity. Until today, electroencephalogram and electromyogram (EEG and EMG) recordings still provide the most accurate data to describe and monitor sleep and wake. At least three major vigilance states can be identified: wake (with low-amplitude high-frequency beta and gamma EEG oscillation between 15-30 Hz and 30-100Hz, as well as extensive EMG activity), non-rapid eye movement sleep (NREM, characterized by large-amplitude delta EEG waves 0.1-4Hz and low or no EMG activity), and rapid eye movement sleep (REM, with predominant theta activity between 6-9Hz and low EMG activity). The relative abundance of these states is governed by both an endogenous 24-hour circadian clock consolidating sleep mostly to day or night, and a sleep homeostat that directs sleep in proportion to the intensity and duration of prior waking experience [3].

Both for the understanding of sleep itself and to study the pathological significance of altered sleep, the identification of individual episodes of sleep and wake across the day based upon EEG/EMG recordings represents a crucial first step. For animals such as rodents EEG/EMG are normally first segmented into 1-8 sec epochs, and are then epoch-by-epoch categorized into the three cardinal vigilance states. Based on the derived scorings, sleep researchers are able to describe the dynamic regulation of sleep, its evolution over time, and the intensity of various EEG oscillations in each vigilance state (its “spectral composition”). Traditionally, this initial classification has been done manually. However, visual inspection-based sleep scoring is a laborious and ambiguous process that requires constant focus of well-trained human experts. Manual sleep scoring is also highly prone to inter-individual variability, and 90-95% agreement in distinguishing vigilance states is usual across human experts (as our study suggests). This data annotation bias is a potential source of inconsistencies between experimental sleep studies.

In past years, numerous approaches have been developed with the aim of automating sleep scoring procedures for human and non-human species. Classical methods transform epochs into hand-designed feature vectors that enable simple discrimination between vigilance states. Manual feature extraction from EEG/EMG is usually based on the domain knowledge already applied in visual sleep scoring. Most popularly, feature vectors are formed from energies of standard frequency bands of EEG power spectrum, such as delta *δ*(0.1–4Hz), theta *θ*(6–9Hz), sigma *σ*(10–15Hz) and beta *β*(15–30Hz) [4–7], or sometimes more fine-grained binning of the spectrum is applied [8–10]. Alternatively, features are extracted directly from the temporal domain [8]. Various machine learning methods are then employed to learn to map derived features to vigilance states i.e. to score sleep. Depending on how these methods utilize annotated data, learning procedures are performed in a supervised [4–6, 9] or unsupervised [7, 10] fashion.

In a wider context, following the first groundbreaking applications of deep neural networks (DNN) to audio [11] and image [12] modeling, DNNs have been very successfully applied to a sequence of related real-life time-series classification tasks such as video activity recognition [13, 14], text classification [15] and automatic speech recognition (ASR) [11, 16–18]. The latter example provides a particularly pertinent analogy to EEG sleep classification: instead of mapping audio to a sequence of words, one maps EEG/EMG to a sequence of vigilance states. Both problems encounter similar challenges related to subject and environmental changes. While in sleep scoring different animals exhibit different oscillatory activity patterns, in ASR audio patterns differ across speakers due to different accents, noise level, recording device or other voice characteristics.

Generalizing from these other domains, primarily from harvesting the discriminative power of end-to-end DNN architectures, another class of sleep scoring methods has recently emerged. DNNs are either trained on top of manually extracted features [8, 19] or the discriminative features are via end-to-end training learned directly from raw EEG/EMG [20–22] or their time-frequency transforms [23]. Popular deep architectures include convolutional neural networks (CNNs) [22], recurrent neural networks (RNNs) [8, 19], the combination of the two [21, 24], or deep belief networks (DBNs) [20] which additionally involve unsupervised pre-training procedure.

Albeit undoubtedly useful to sleep researchers for (semi-)automated sleep scoring, the prediction accuracy of existing methods is still not equal to that of human experts. Even more importantly, current animal sleep scoring solutions have major difficulties to generalize pattern recognition across animals from different experimental settings and labs. Cross-subject variations in EEG/EMG sleep patterns normally originate from experimental differences such as signal-to-noise ratio, EEG derivation and electrode placement, genetic background, drug application, disease models, or differing lab strains and animal species e.g. rats vs. mice [25]. Due to these signal variabilities, designing robust features by hand is an extremely challenging task. Contrary to approaches attempting to manually construct consistent features, end-to-end architectures have potential to learn robust discriminative features de novo. However, to our knowledge, end-to-end learning frameworks were applied only in the context of human sleep, and furthermore reported solutions showed no significant improvements over the classical hand-derived feature based methods [20, 21].

In this paper, we have devised a novel framework—SPINDLE (Sleep Phase Identification with Neural networks for Domain-invariant LEearning) drawing some inspiration from the traditional hybrid DNN-HMM models [11] which combine deep neural networks (DNN) and hidden Markov models (HMM) in automatic speech recognition (ASR) [11]. In particular, we operated on time-frequency domain and used similar preprocessing procedures to the ones used for extracting Mel frequency cepstrum coefficients (MFCC) [17], generalized to multiple heterogeneous channels. Our approach to solving time-frequency fluctuations using a CNN is also motivated by similar modeling ideas in ASR for dealing with speaker and environmental variability [26]. Finally, we used a hidden Markov model (HMM) to describe vigilance state dynamics and suppress physiologically impossible transitions, similarly to DNN-HMM ASR systems which constrain the output space alleviating infeasible language constructions. SPINDLE overcomes aforementioned performance and generalization issues in automatic sleep scoring for animals: the model was trained only on two wildtype mice, and was then evaluated on 12 mice and 8 rats from four animal cohorts of three independent labs, rendering accuracies of 99%, 98%, 93% and 97% in signal areas where human experts agreed. When compared to the individual human experts, SPINDLE showed practically equal agreement rate to the one human experts had between themselves, both for artifacts and vigilance states. Specifically, our main contributions are:

1. Through the spectral profile analysis we explain why current solutions do not generalize well across experimental domains without additional parameter calibration. This severely limits their applicability. We then elaborate why the proposed CNN-HMM architecture is suitable for this task.
2. We present SPINDLE—a novel ASR-inspired computational method for fast, accurate and physiologically plausible sleep scoring in animals. In terms of predictive performance, our method is vastly superior to existing ones and is fully comparable to trained human experts.
3. We disclose a diverse double scored data set (14 mice and 8 rats, annotated by five human experts, rendering 950.400 labels in total) assembled from EEG/EMG recordings produced in three separate sleep labs. The data may be used in the future for benchmarking purposes.
4. By validating the performance of SPINDLE on the collected data, we demonstrate that without any re-training or fine tunning, our model achieves high predictive accuracy on subjects with different EEG/EMG characteristics, originating from different experimental conditions, labs, and even species.
5. Furthermore, we show that SPINDLE is capable of identifying statistically significant alterations of sleep patterns. We illustrate this by comparing a wild and the corresponding genetically modified mice strain, deriving the same conclusions as scores by two independent human experts.
6. We contribute with a publicly available free web service for simple and quick classification of EEG/EMG animal recordings.

### SPINDLE method overview

**Fig 1:**
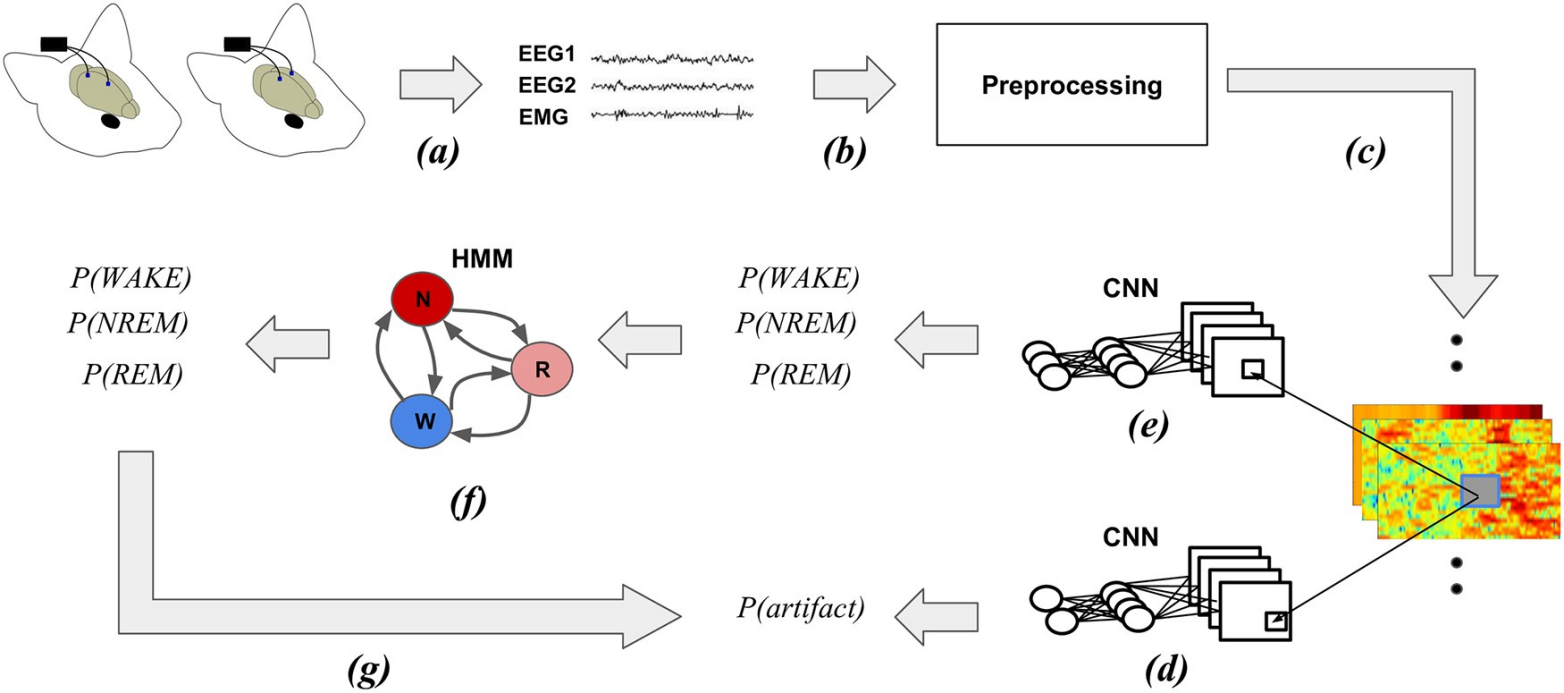
Conceptual overview of the SPINDLE framework. **(a)** Measured EEG activity may vary depending on where the electrodes are placed. Assumed input in our setting are two EEG channels and one EMG channel. EMG signal is recorded on the neck muscle (not depicted for simplicity). **(b)** Raw signals are processed by windowed Fourier transforms applied on overlapping frames. The output of the preprocessing are time-frequency representations of EEG/EMG which are additionally preprocessed. **(c)** Three two-dimensional spectrograms are then sectioned into epochs which correspond to 4 sec intervals. Each epoch is independently processed by the two CNNs. **(d)** The first CNN estimates whether the evaluated epoch is an artifact. **(e)** The second CNN estimates the probability of each vigilance state. **(f)** The sequence of estimated vigilance state probabilities is then corrected using the Viterbi decoding algorithm and predetermined transition matrix of HMM which encodes the transition rules. If an epoch is not designated as an artifact, the most probable vigilance state is assigned. **(g)** If an epoch is marked as an artifact, the most probable vigilance state determines the type of the artifact: WAKE-artifact, NREM-artifact or REM-artifact. NREM/N, non-rapid eye movement; REM/R, rapid eye movement; WAKE/W, wakefulness; CNN, convolutional neural network; HMM hidden Markov model.

The SPINDLE method presented here (sketched in Fig 1) is designed to achieve high predictive performance preserved across different experimental settings and labs. Its architecture is carefully crafted in an end-to-end fashion around a convolutional neural network (CNN) which operates on top of the preprocessed time-frequency channels of EEG/EMG. We exploit the ability of the CNN to learn highly discriminative and translation-invariant features, as this allows us to remain agnostic to changes in sleep patterns, in both time and frequency dimension. On top of the CNN, a hidden Markov model (HMM) describes vigilance state transition dynamics and suppresses physiologically infeasible vigilance state transitions when applicable. To account for artifacts, SPINDLE contains an additional CNN with binary output: artifact or non-artifact, which is combined with the predictions of vigilance states to determine the artifact types (steps (d) and (g) in Fig 1 respectively). For a detailed explanation, please refer to the Materials and Methods section further below.

SPINDLE was tested on data produced in three independent sleep labs: BrownLab (www.sbrownlab.com), TidisLab (http://tidis-lab.org/) and BaumannLab (http://www.sleep.uzh.ch/en/research-groups/group-baumann.html). The collected data consists of a number of rodent EEG/EMG recordings acquired during sleep studies performed with varying experimental paradigms. The recordings were clustered into four animal cohorts with similar characteristics as summarized in Table 1, and evaluated separately.

**Table 1:** Collected data overview. Presented are the notable properties of EEG/EMG animal recordings produced in our study. All recordings were segmented into 4 sec time intervals (epochs) and then annotated rendering 21600 × 2 labels per animal. Table columns for each cohort and lab depict: (a) the number of wildtypes; (b) the number of mutants; (c) rodent specie; (d) the sampling rate of the recording device; (e) the derivation of 2 EEG signals with respect to the placement of corresponding EEG electrodes; (f) the derivation of EMG signal; (g) the number of human experts who scored the data; (h) the duration of each animal recording within given cohort; and lastly (i) the degree of signal corruption taken as the average percentage of artifacts computed from the scorings of the corresponding experts. The cohort C was scored by an expert from BaumannLab, as well as by an expert from BrownLab. All other cohorts were scored by experts from the same lab. Data acquisition is for each animal cohort explained in detail in Materials and Methods.

Cohort	Lab	Wild	Mutant	Specie	S.Rate	EEG Derivations	EMG Derivation	Experts	Duration	Artifacts
A	BrownLab	4	0	mice	128Hz	1 frontal, 1 parietal	neck	2	24h	15.2%
B	BrownLab	0	4	mice	128Hz	1 frontal, 1 parietal	neck	2	24h	19.2%
C	BaumannLab	8	0	rats	200Hz	2 parietals	neck	1+ 1	24h	21.3%
D	TidisLab	6	0	mice	512Hz	1 frontal, 1 parietal	neck	2	24h	≈0%

## Results

### Analysis of scoring variability across human experts

**Fig 2:**
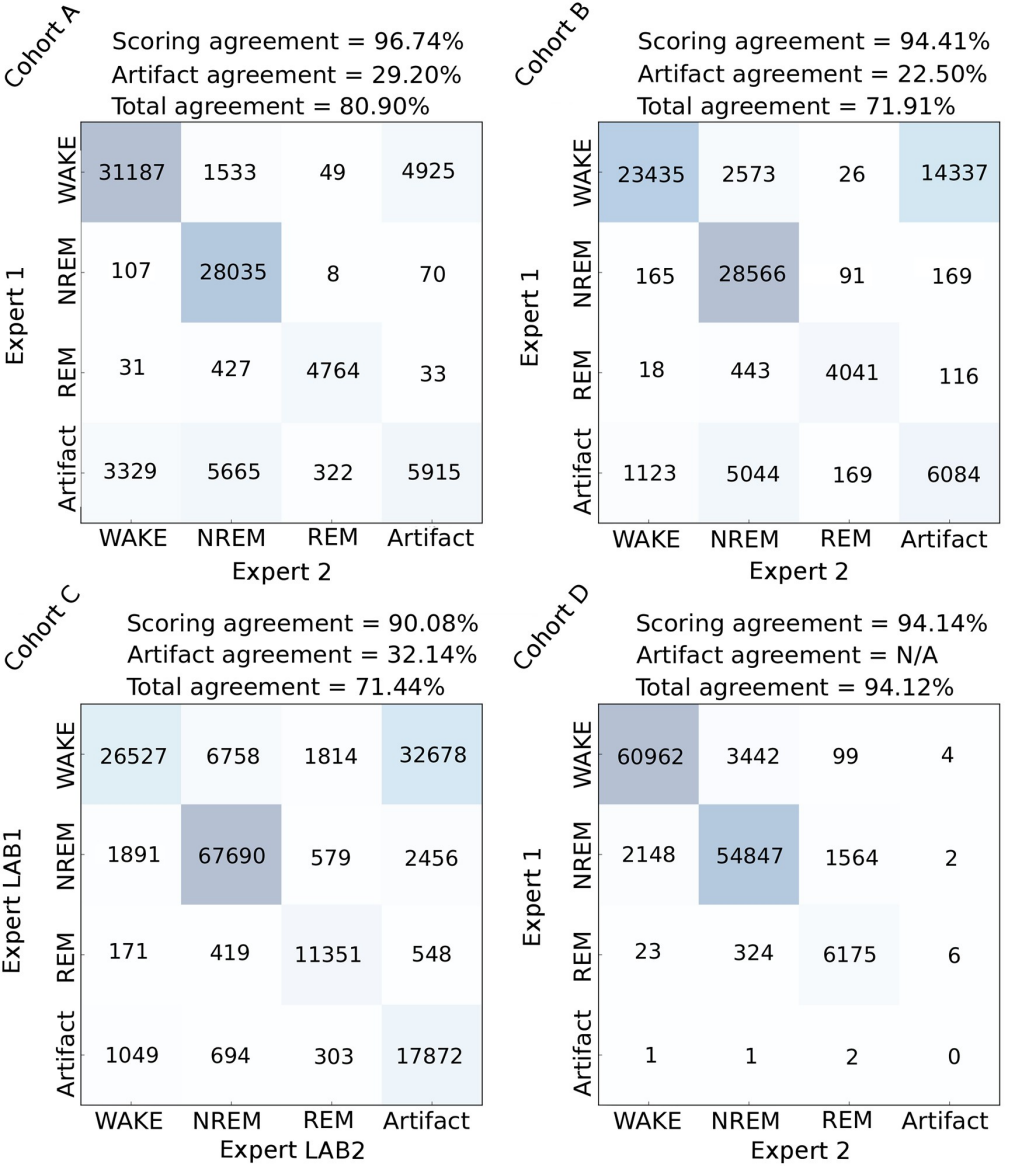
Intra-lab and inter-lab human expert agreement. Confusion matrices derived from the twofold annotation procedure of EEG/EMG data with number of common epochs shown at each intersection, and overall percentage agreement calculated above. We evaluated the agreement of human experts from the same lab (intra-lab agreement), but we also compared the scorings of a BrownLab human expert with the scorings of a BaumannLab human expert on the cohort C (inter-lab agreement). The agreements were computed per-cohort, for non-artifact and artifact data separately, and again when taking all epochs into account.

One of the major issues of visual inspection is the intrinsic subjectivity of human experts in data annotation, especially in ambiguous cases when signal patterns do not clearly adhere to the predefined scoring rules. For example, during the transition between vigilance states, it is not always clear where the actual state change occurs. Taking this into consideration is particularly relevant for the validation of an automated sleep scoring method. To this end, we analyzed the inter-expert scoring agreement in evaluation of identical EEG/EMG data (see Fig 2). To estimate the coherence between human experts, we first measured their agreement in regions that no expert identified as “artifacts”—EEG/EMG perturbations related to environmental interference rather than changes in brain state. We then computed the accuracy from the corresponding 3 × 3 vigilance state submatrices from Fig 2. When comparing human experts from the same lab, the estimated agreement rate of sleep scoring in non-artifact data was 95-96%, while the inter-lab agreement was about 90%. On the other hand, the disagreement between human experts in classification of artifacts was notably higher, as the figure indicates. To measure this, we calculated the ratio between the number of epochs marked as corrupted by both experts and the number of epochs marked as corrupted by at least one of the two experts:

$$\begin{array}{c} \begin{array}{c} artifact\_scoring\_agreement=\frac{\left|artifact\_intersection(expert_{1},expert_{2})\right|}{\left|artifact\_union(expert_{1},expert_{2})\right|} \end{array} \end{array}$$ (1)

These numbers provide rough estimates of the expected accuracy bounds of a hypothetical sleep scoring method comparable to human experts in terms of the predictive performance.

### Analysis and adaptation to variability of spectral profiles

**Fig 3:**
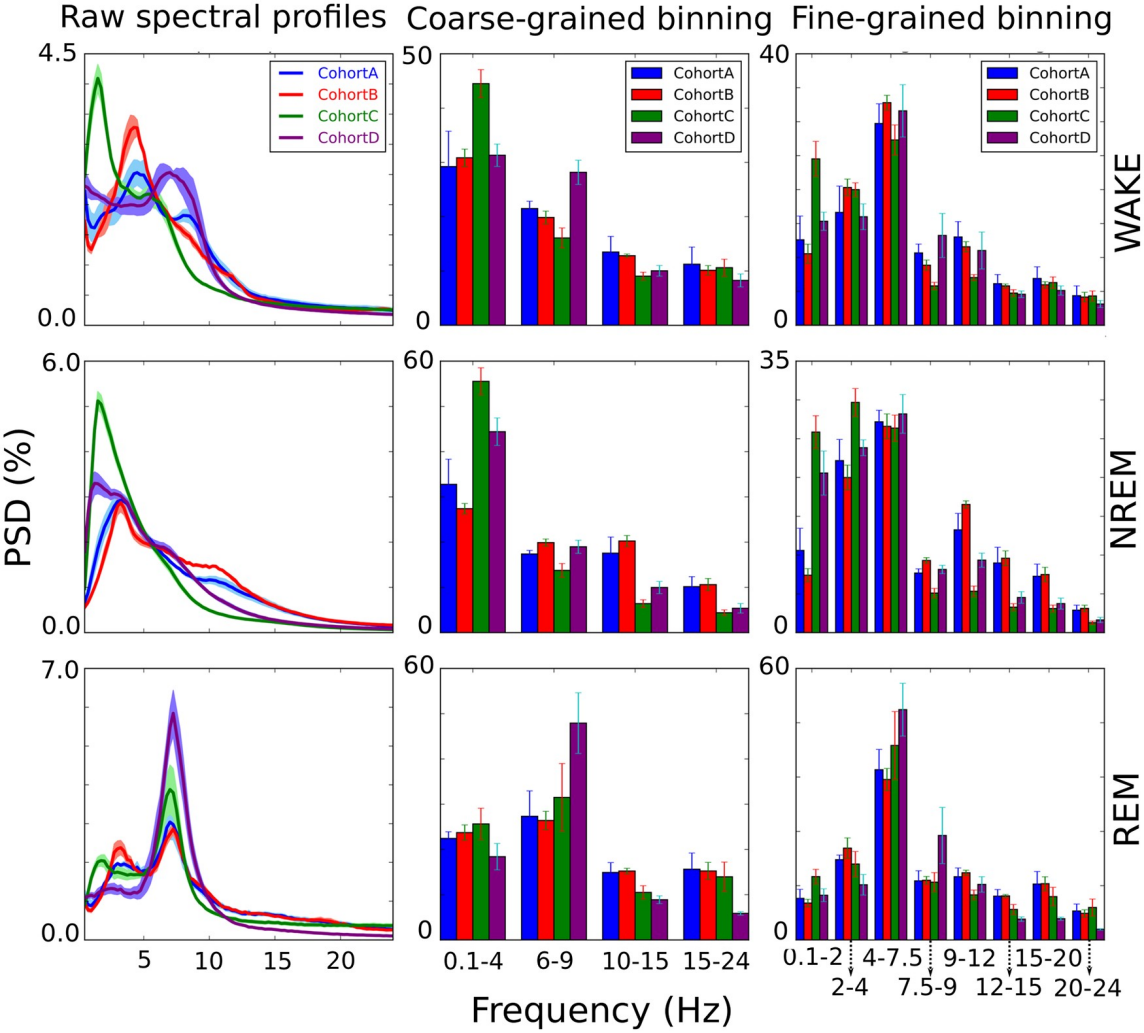
Spectral profiles. For each animal, the averaged frequency spectrum of the EEG recording (its spectral profile) was computed per vigilance class. Each plot in the left column is related to one of the 4 animal cohorts and consists of the mean spectral profile curve and the corresponding standard deviation (a half of it). All curves are normalized relative to the total power of the signal. The middle and the right column respectively represent coarse-grained (following the classical delta, theta, sigma and beta bands) and fine-grained histogram binning applied to the raw spectral profiles, with bars representing summed spectral power in each bin.

To identify the key obstacles towards robust cross-subject sleep classification, we analyzed the fluctuations of EEG in epochs classified as belonging to the same vigilance state. In the context of our problem, ideally, for each vigilance state we would have signal patterns which are consistent (i) across epochs within the same subject; (ii) across subjects within the same animal cohort; (iii) across subjects from different animal cohorts analyzed under different experimental conditions. To explore the variability across these categories, for each animal and for each vigilance state we separately averaged EEG frequency spectra over all epochs, and then compared these measures within and across cohorts (Fig 3). Whether we applied coarse-grained histogram binning according to the commonly used frequency bands (Fig 3, middle column) or finer binning (Fig 3, right column), spectral energy was differently distributed among frequency bands for different animal cohorts. For example, even though the figure indicates the existence of certain patterns in sleep state signatures i.e. a prominent peak at around 7Hz characteristic of REM sleep, this cannot be simply interpreted as a rule due to high cross-epoch, cross-animal and cross-cohort variabilities [25, 27]. This is arguably the main reason why the classical methods which base their features on energies of different frequency bands of power spectrum do not generalize well. The feature vectors of equal vigilance states are highly inconsistent across subjects, especially if animals have significantly different backgrounds.

**Fig 4:**
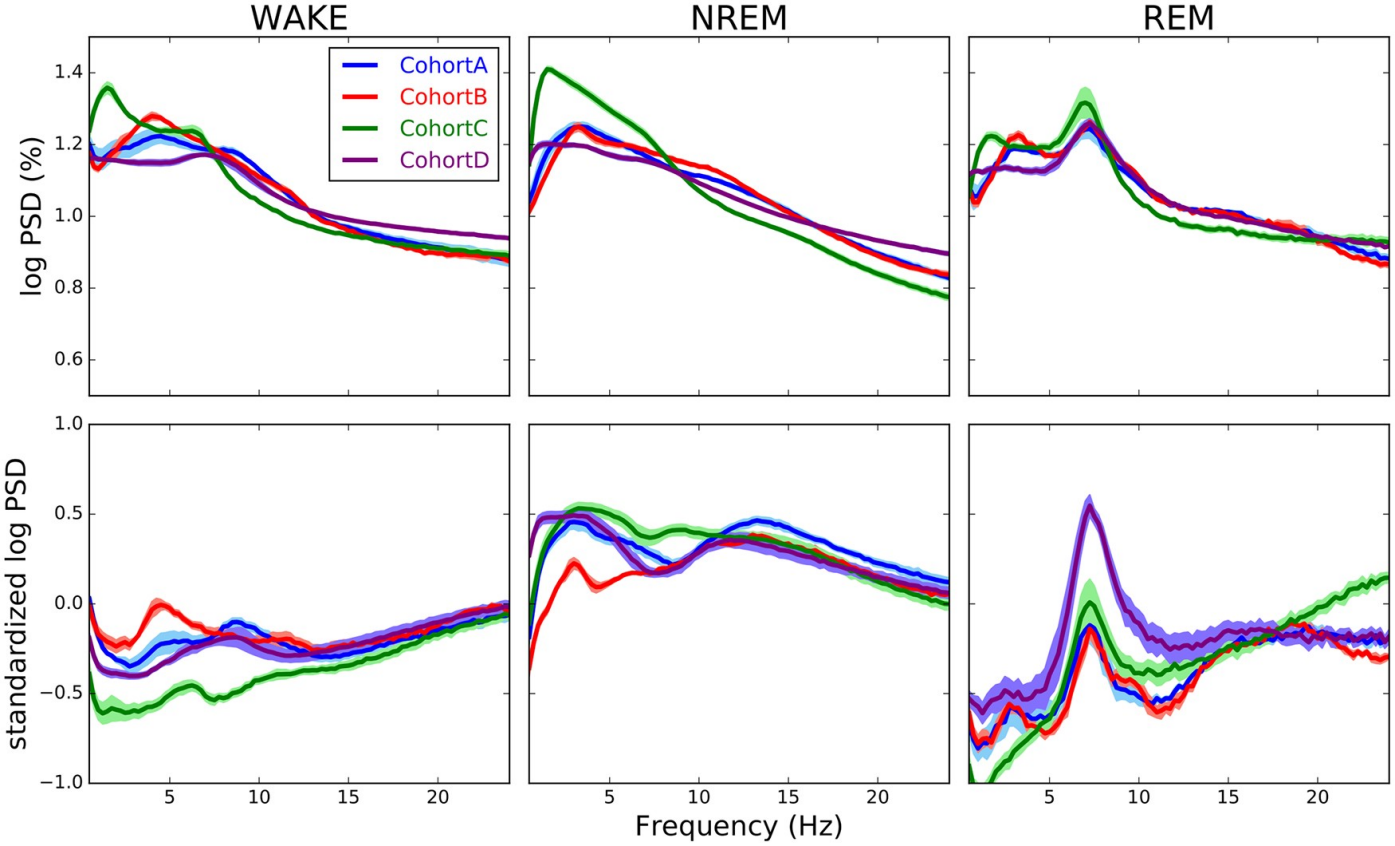
Effects of preprocessing to spectral profiles. Top row shows per vigilance state spectral profiles normalized relative to the total signal power, but only after the log transformation was applied. Relative differences in amplitudes between cohorts are attenuated (compared to Fig 3). Bottom row shows log transformed curves after being per frequency component standardized to emphasize the differences between vigilance states.

To overcome these variations, SPINDLE employs a preprocessing procedure to increase the consistency of spectral patterns within the samples of the same vigilance class. The effects of preprocessing are illustrated in Fig 4. On one hand, the log transformation attenuates the discrepancies in magnitudes, and on the other the typical zero mean/unit variance standardization emphasizes the differences between vigilance states. The core characteristic of SPINDLE, however, is its ability to adapt to the variations of sleep state pattern variations in the frequency axis. This flexibility is achieved through translational invariance, an intrinsic property of CNNs. Whenever the frequency spectrum of evaluated data sample deviates from a hypothetically expected spectral pattern in terms of small shifts of relevant peaks, the CNN absorbs these shifts through the convolutional and max-pooling layers (see Materials and methods).

### SPINDLE—Qualitative analysis

**Fig 5:**
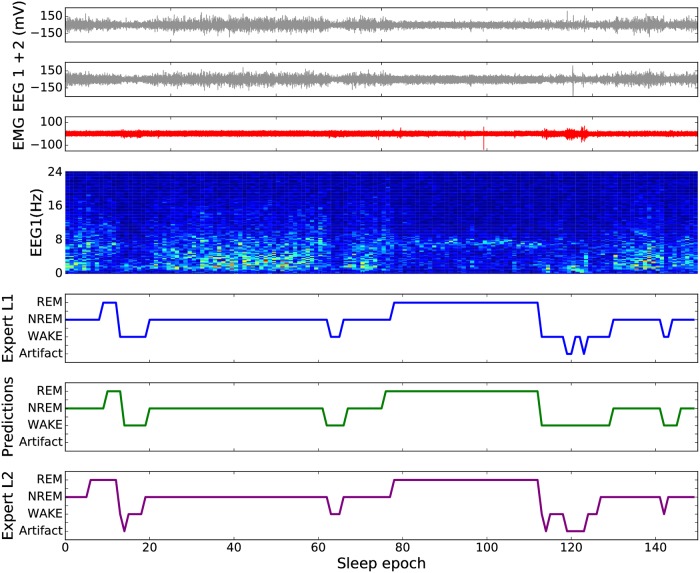
Qualitative analysis of SPINDLE. 150 epochs were extracted from an animal from the cohort C, where we found the automated classification to be more challenging, to qualitatively compare the predictions of SPINDLE to the scorings of two experts from different labs. The first three signals from top represent the input, two EEG and an EMG. The spectrogram in the middle is a time-frequency representation of one of the EEG signals. The bottom three plots are the hypnograms, the first one derived from the scorings of one human expert, the second one derived from the predictions of SPINDLE and the third one derived from the scorings of the other human expert.

Before providing a rigorous statistical performance evaluation of SPINDLE, we illustrate its general applicability in Fig 5, where we visually compare the scorings of two human experts from different sleep labs with the predictions of our method on identical portion of an EEG/EMG recording from the cohort C. The figure shows that the agreement between the predictions of SPINDLE and the corresponding experts is visually appealing and also sheds light on some common sources of disagreements in the sleep scoring procedure. When vigilance state is constant, the predictions are mainly in agreement, but during the transitions between vigilance states, disagreements are frequent both between human expert scorers and between human and automatically generated scorings. The figure also shows that artifacts are another common source of disagreements. Finally, it is illustrated why time-frequency representation is useful for understanding sleep dynamics i.e. it is easy to notice the correlation between the spectral patterns in the spectrogram and the corresponding vigilance states.

### SPINDLE—Quantitative analysis

In a comprehensive quantitative study we evaluated different performance aspects of SPINDLE. For this purpose, the data set (previously summarized in Table 1) was separated into training and testing subsets. There was no overlap between training and testing sets in any of our experiments. The training set consisted of 2 wildtype mice taken from the cohort A, while the validation was performed on the rest of the data i.e. 20 remaining rodents from different labs, strains, and species. Splitting the data set in this way enabled us to test the main premise of this paper: the robustness of our method holds for different experimental settings and labs without any additional model adaptation. By training SPINDLE only on wildtypes we were able to investigate how well it generalizes across the subjects of the same kind (two other wildtypes from the same cohort A), genetically mutated animals (4 mice from the cohort B), different animal species (the rats from the cohort C), and different sleep labs (comparing across cohorts A/B, C, and D).

First, to diminish the effect of subjectivity in manual sleep scoring, we evaluated the predictions against human expert scoring intersection—epochs in which two human experts agree on the label, since all animal recordings were double scored by two individuals. Here, the epochs in which two human experts disagreed did not have any influence upon the performance evaluation. Secondly, to avoid discarding hard-to-score signal regions, we additionally analyzed the performance with respect to the human experts individually. Finally, as we mention above, artifacts represent a major source of difficulty for human expert scorers (recall Fig 2). To evaluate SPINDLE more accurately in this respect, we then considered “clean” regions and artifacts separately.

More concretely, we first measured the agreement of vigilance state predictions (given by the output of step (f) in Fig 1) with the corresponding human expert scorings only for the epochs not marked as artifacts (by any of the two human experts). To evaluate the quality of artifact detection, the 4-category scorings were re-labeled into non-artifacts and artifacts, and then compared to the corresponding predictions (given by the output of step (d) in Fig 1). In addition, we investigated the benefits of applying hidden Markov model (HMM) based post-processing, comparing the predictions of HMM (the output of step (f) in Fig 1) against the predictions of the convolutional neural network (CNN) (the output of step (e) in Fig 1). Evaluations were conducted according to the usual classification metrics, first of overall Accuracy *AC*, and then for each vigilance state in terms of Precision *PR*^(*C*)^, Recall *RC*^(*C*)^ and F1-score *F*1^(*C*)^ for each vigilance state C, in percentages:

$$\begin{array}{c} \begin{array}{ccc} AC & =\frac{TP+TN}{\#samples}\cdot 100 & \;PR^{\left(C\right)}=\frac{TP^{\left(C\right)}}{TP^{\left(C\right)}+FP^{\left(C\right)}}\cdot 100\\ RC^{\left(C\right)} & =\frac{TP^{\left(C\right)}}{TP^{\left(C\right)}+FN^{\left(C\right)}}\cdot 100\; & F1^{\left(C\right)}=\frac{2\cdot PR^{\left(C\right)}\cdot RC^{\left(C\right)}}{PR^{\left(C\right)}+RC^{\left(C\right)}}\cdot 100 \end{array} \end{array}$$ (2)

where *TP*^(*C*)^, *FP*^(*C*)^ and *FN*^(*C*)^ are for vigilance class *C* the numbers of true positives, false positives and false negatives respectively. *TP* and *TN* represent the total number of true positives and true negatives.

Table 2 demonstrates the predictive performance of SPINDLE with respect to these metrics (excluding artifacts as described above). We compared the predictions of SPINDLE against the scoring intersection of corresponding human experts. The evaluation was performed with and without HMM-based postprocessing which additionally enforced physiological constraints on vigilance state transitions. Although CNN generates impressive performance alone (top rows), HMM leads to additional improvements (bottom rows), most notably in identification of the REM phase which is usually more challenging to score [10]. SPINDLE hence showed that injecting sleep domain knowledge into the model may induce very positive effects on the predictive performance. Across all wildtype and mutant mouse species and across labs, overall accuracies exceeded 97%. Across species, overall accuracy remained at 93%, demonstrating the generalization capabilities of SPINDLE.

**Table 2:** Predicting vigilance states—Agreement analysis. The evaluation was performed with and without the application of HMM based post-processing (the outputs of steps (f) and (e) in Fig 1 respectively). The predictive power is quantified with respect to the global accuracy, and for each vigilance state separately with respect to the precision, recall and F1-score according to Eq 2.

CNN predictions		WAKE			NREM			REM		
Cohort	Accuracy	Precision	Recall	F1-Score	Precision	Recall	F1-Score	Precision	Recall	F1-Score
A	99 ± 0.5%	100 ± 0.3%	99 ± 0.4%	99 ± 0.3%	99 ± 0.8%	99 ± 0.4%	99 ± 0.6%	98 ± 1.6%	97 ± 2.6%	98 ± 1.2%
B	98 ± 0.3%	98 ± 2.1%	97 ± 1.1%	98 ± 0.7%	97 ± 0.9%	98 ± 1.1%	98 ± 0.1%	96 ± 2.2%	95 ± 1.9%	96 ± 0.9%
C	92 ± 3.2%	80 ± 10.0%	97 ± 1.5%	87 ± 6.0%	99 ± 1.0%	94 ± 3.4%	96 ± 1.8%	86 ± 5.2%	70 ± 16.8%	76 ± 12.2%
D	97 ± 1.3%	98 ± 1.0%	99 ± 0.7%	98 ± 0.6%	97 ± 2.7%	98 ± 1.1%	97 ± 1.2%	96 ± 3.5%	79 ± 23.0%	85 ± 17.7%
CNN+HMM		Wake			NREM			REM		
Cohort	Accuracy	Precision	Recall	F1-Score	Precision	Recall	F1-Score	Precision	Recall	F1-Score
A	99 ± 0.4%	100 ± 0.3%	99 ± 0.4%	99 ± 0.3%	99 ± 0.8%	99 ± 0.4%	99 ± 0.6%	98 ± 1.5%	97 ± 2.5%	98 ± 1.1%
B	98 ± 0.4%	98 ± 2.1%	97 ± 1.0%	98 ± 0.7%	97 ± 0.9%	98 ± 1.1%	98 ± 0.1%	97 ± 2.4%	96 ± 2.0%	96 ± 0.8%
C	93 ± 3.4%	81 ± 10.6%	98 ± 1.5%	88 ± 6.4%	99 ± 1.0%	94 ± 3.6%	96 ± 1.8%	94 ± 3.0%	75 ± 17.7%	82 ± 13.0%
D	97 ± 1.3%	98 ± 1.0%	99 ± 0.8%	98 ± 0.6%	97 ± 2.8%	98 ± 1.0%	97 ± 1.2%	97 ± 3.3%	81 ± 23.0%	86 ± 17.0%

**Fig 6:**
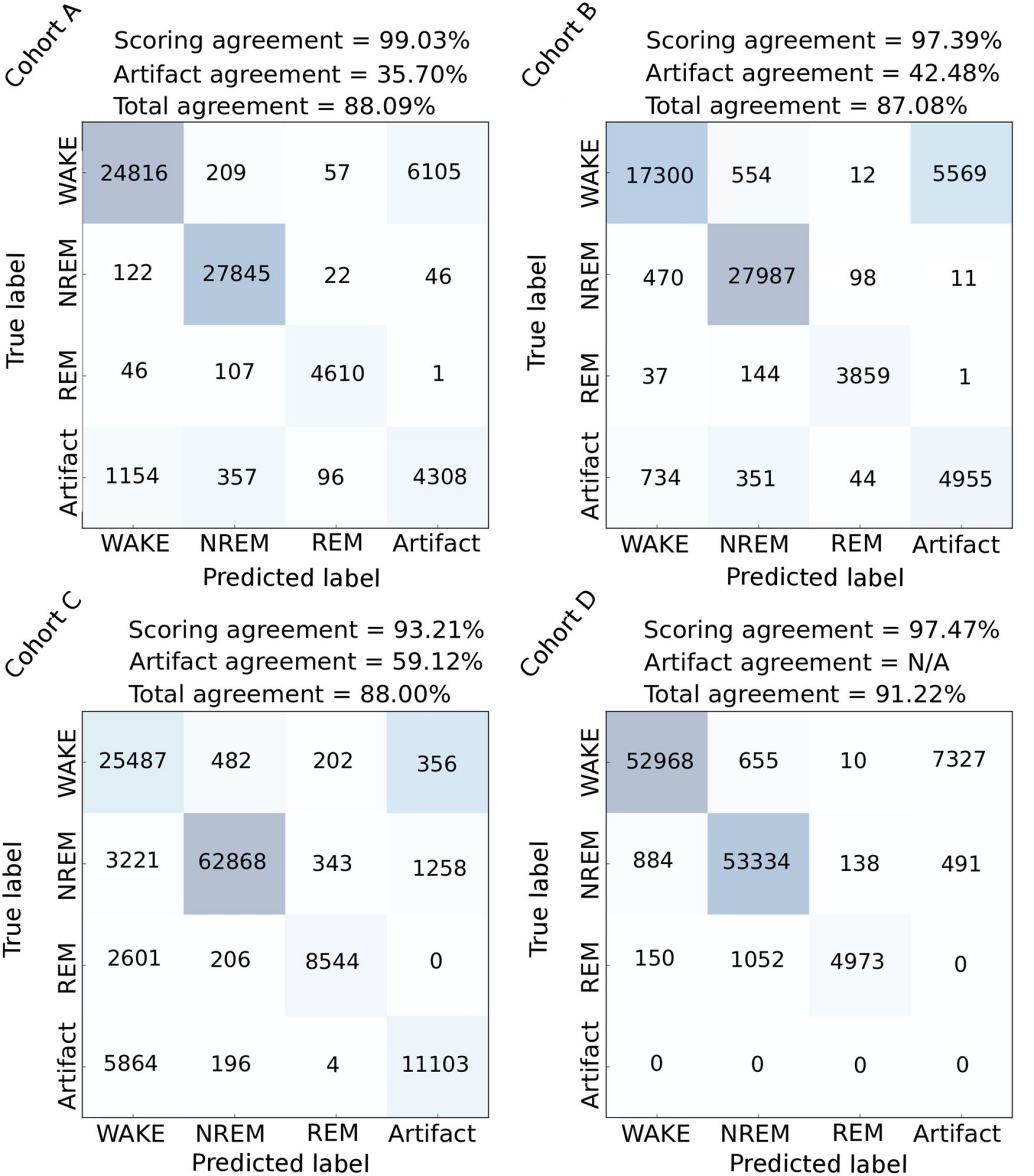
Comparison against expert intersection—Confusion matrices. Our predictions were for each cohort independently compared to the annotation intersection of two human experts. Presented are the corresponding confusion matrices. The total and vigilance state scoring agreement was calculated with respect to Eq 2, whereas the artifact scoring agreement was calculated as described in Eq 1.

The corresponding confusion matrices computed by comparing the label intersection of two human experts to our predictions are given in Fig 6 and show how relatively few mislabeled epochs are confused across different vigilance states. For completeness sake, the figure also shows the confusion with respect to artifact identification. The final agreement calculated when artifacts are taken into account is presented in addition.

Next, we compared the predictions of SPINDLE against the scorings of individual human experts. Doing so enabled us to (i) include into our analysis the epochs in which two experts disagreed; (ii) investigate the potential of SPINDLE to generate predictions which are indistinguishable from the scorings produced by human experts: ideally, the agreement between a human expert and SPINDLE would be close to the agreement between two human experts. The results of the analysis given in Table 3 are more than encouraging and show that in terms of the global scoring agreement i.e. the accuracy, SPINDLE is perfectly comparable to human experts. The agreement rate between the predictions of SPINDLE and each expert is close to the agreement rate between two corresponding experts.

**Table 3:** Predicting vigilance states—Comparison against individual human experts. The table shows global agreement rate measured by comparing (a) individual experts between themselves; (b) individual experts with SPINDLE; (c) the scoring intersection of two experts with the predictions of SPINDLE. The evaluation was performed on each cohort separately and only non-artifactual epochs were taken into account.

	Cohort			
	A	B	C	D
Expert 1 vs Expert 2	96.9%	94.5%	90.0%	94.1%
Expert 1 vs SPINDLE	97.7%	96.0%	89.5%	94.9%
Expert 2 vs SPINDLE	96.7%	93.7%	88.7%	94.5%
Expert intersection vs SPINDLE	99.3%	98.1%	92.8%	97.4%

Finally, in a separate set of experiments we evaluated the predictive performance of SPINDLE in identifying artifacts. The subjectivity in distinguishing artifacts from clean epochs is generally known to be overwhelming, and similar conclusions may be derived from Fig 2. To ensure a fair performance estimation we computed the agreement rate with each human expert separately, and then compared it to the agreement rate between the two human experts. Table 4 suggests that SPINDLE’s predictions are, in terms of global agreement as defined by Eq 1, de facto equal to that of human experts.

**Table 4:** Predicting artifacts—Comparison against individual human experts. The table shows global agreement rate in artifact detection (evaluated with respect to Eq 1) by comparing (a) individual experts between themselves; (b) individual experts with SPINDLE; (c) only the epochs marked as artifacts by both experts to the artifact predictions of SPINDLE. Note that the cohort D was omitted since it contained practically no epochs labeled as artifacts.

	Cohort		
	A	B	C
Expert 1 vs Expert 2	29.2%	22.5%	32.1%
Expert 1 vs SPINDLE	24.6%	19.5%	40.3%
Expert 2 vs SPINDLE	36.1%	51.8%	32.9%
Expert intersection vs SPINDLE	35.7%	42.5%	59.1%

### Comparison to other approaches

We compared SPINDLE to three previously reported approaches, using the same data identically split into training and testing sets as already described. Analysis was performed only on the epochs where human experts agreed on the label. Since other algorithms lack dedicated artifact detection and analysis subroutines, corrupted epochs were not taken into account. The following three methods were used as our baselines:

#### FASTER [10]

An unsupervised learning approach which uses nonparametric density estimation clustering on top of manually extracted features. The features in FASTER are derived from a comprehensively binned EEG/EMG power spectra, and are further compressed via principal component analysis. The training data were used for optimizing the hyperparameters according to the procedure described by the authors. Since FASTER was originally applied to 8 seconds long epochs, we down-sampled our scoring resolution from 4 to 8 seconds and then kept only the new larger epochs which contained two equal labels. This way we ensured that FASTER is not at any disadvantage due to the different data annotation setup, furthermore even giving it some advantage by discarding many of the state transition epochs which are hard to score.

#### SCOPRISM [6]

SCOPRISM uses two features: (i) the ratio between EEG spectral power of theta *θ*(6-9Hz) and delta *δ*(0.5-4Hz) frequency ranges; (ii) the root mean squared error of the EMG signal. The two-dimensional feature space is separated according to threshold values which are learned from data. The sleep scoring of each epoch is further refined, following the results of the scoring draft in adjacent epochs. We optimized the thresholds with respect to the training set and kept them fixed during the evaluation on the testing set. SCOPRISM is originally designed for 4 seconds epochs thus no further parameter adaptation was required.

#### Autoscore [9]

This method extracts features from power spectra with respect to the logarithmic distribution. Classification in Autoscore is performed using naive Bayes classifier. The feature vectors are smoothed time-wise using a Gaussian convolution to reduce noise. The method eliminates epochs using a 1-40Hz band-pass filter of the EMG signal. This artifact removal approach produced poor overlap with the artifacts from our data set so it was not further considered. The method was set to produce sleep scoring predictions for 4 second epochs.

Since the competing methods were designed to operate on 1EEG/1EMG setup (rather than 2EEG/1EMG), for each approach we explored the following variations: (a) using only the first EEG signal; (b) using only the second EEG signal; (c) using the average of two EEG signals; (d) averaging the features extracted from two EEG signals; (e) forming the feature vector by combining the features extracted from two EEG signals. In each case, the alternative performing best for the other algorithm was used in our analysis.

**Fig 7:**
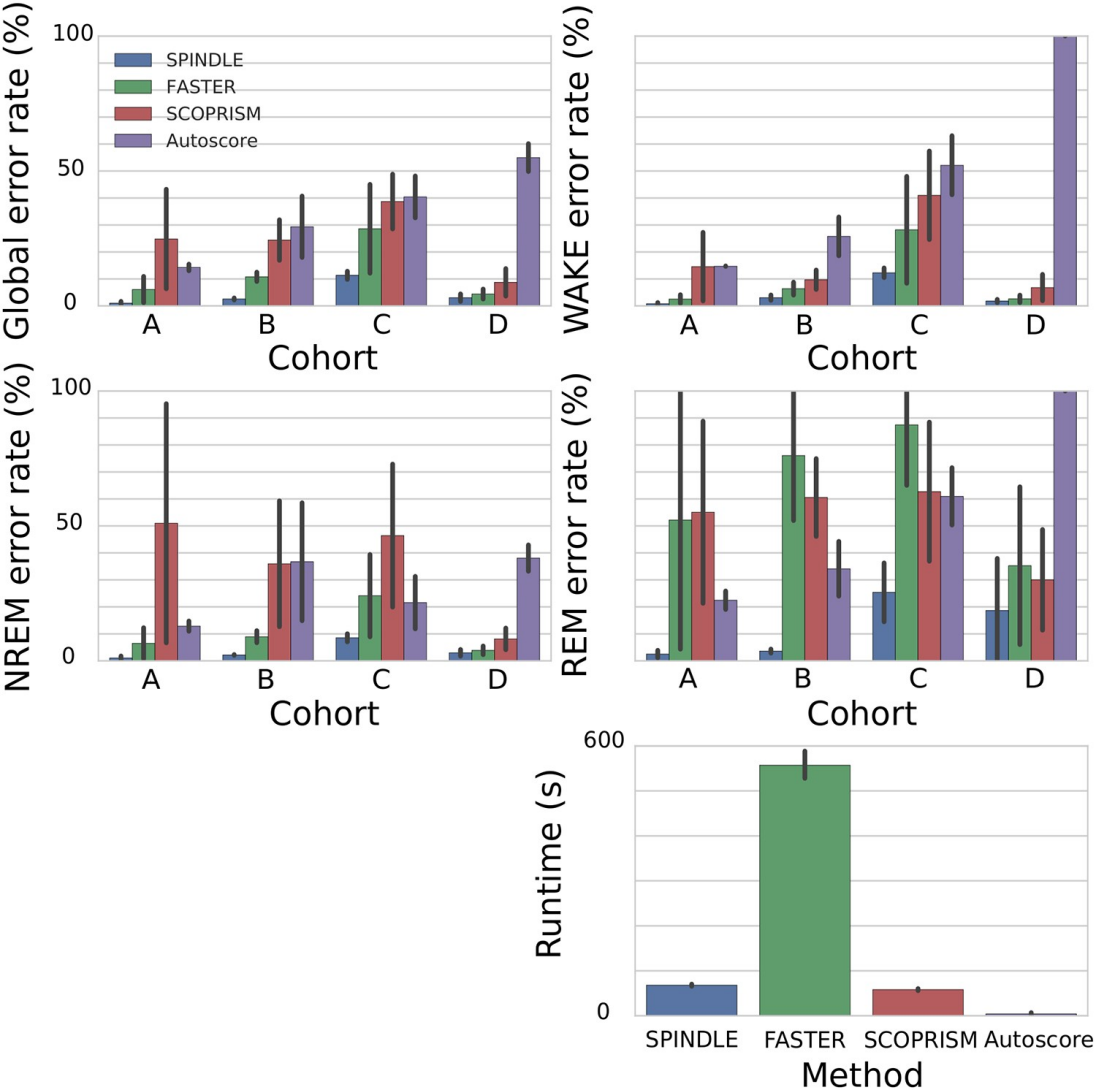
Comparative analysis of SPINDLE. SPINDLE was compared against three other state-of-the-art solutions (FASTER [10], SCOPRISM [6] and Autoscore [9]). Evaluations were performed for each animal separately and the results were grouped per cohort (top four figures). The global error rate was measured as *ER* = 100 − *AC*, and for each vigilance state separately the class specific error rate was measured as *CER*^(*C*)^ = 100 − *F*1^(*C*)^, where *AC* and *F*1^(*C*)^ are defined in Eq 2. The evaluation of errors was performed on the scoring intersection of two human raters and did not take corrupted epochs into account. Execution times for scoring of 24 hour EEG/EMG animal recordings are given at the right bottom figure.

Fig 7 presents the summary of the comparative analysis. The experiments showed remarkable reduction in both—the error rates and the corresponding standard deviation, which indicates that SPINDLE is much more accurate, and furthermore is more robust than the previous solutions. Particularly appealing is its superiority in detecting REM sleep which is generally considered more difficult to identify. It is also worthwhile considering the computational intensity of the competing methods (bottom right side of Fig 7). SPINDLE provides impressive predictive performance in a reasonable time frame.

**Fig 8:**
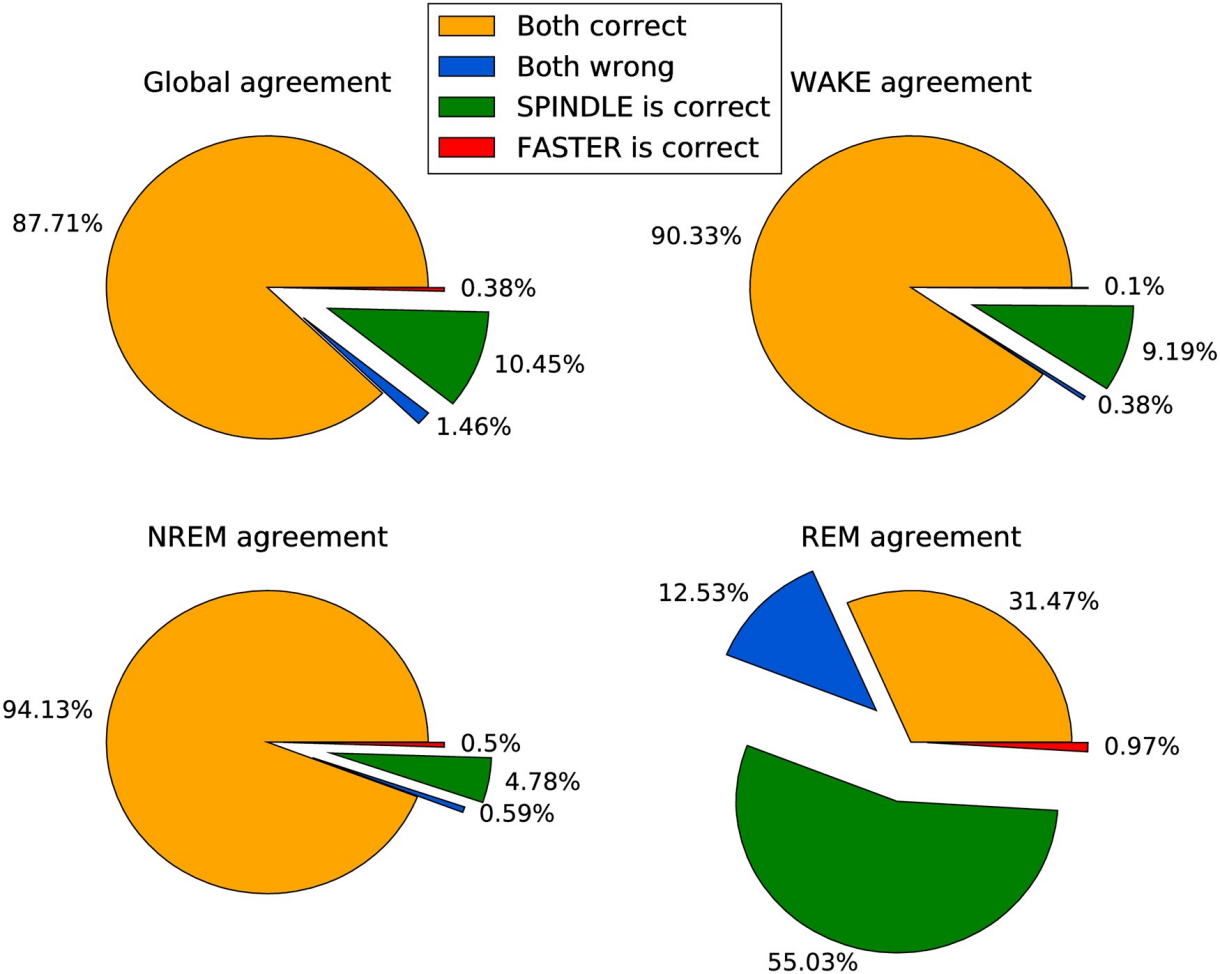
Scoring agreement comparison of FASTER and SPINDLE on 8 second intervals. The overlap was measured with respect to all epochs from all animal cohorts, and additionally with respect to each vigilance state individually.

Furthermore, we analyzed the prediction overlap between SPINDLE and FASTER, to understand their agreement with respect to the ground truth annotations. The analysis is given in Fig 8. Note again that in order to ensure a consistent comparison, we down-sampled ground truth and SPINDLE’s predictions to 8 second epochs, and discarded epochs which contained: (i) artefacts; (ii) inconsistent scoring between human experts; (iii) label confusion as a product of down-sampling.

### SPINDLE—Output analysis

After epoch-by-epoch classification, sleep data is typically pooled across each vigilance state, and then quantitatively evaluated with respect to various parameters of sleep architecture, sleep timing and EEG spectral power. Therefore, we next compared such quantitative outputs when calculated using classifications from SPINDLE or from human scorers. To that end, we analyzed the performance of the downstream analysis applied to the predictions of SPINDLE to investigate its capability to (i) predict major parameters of the sleep architecture; (ii) detect sleep alteration induced by a genetic mutation performed on the cohort B, with respect to the cohort A.

#### Sleep architecture analysis

**Fig 9:**
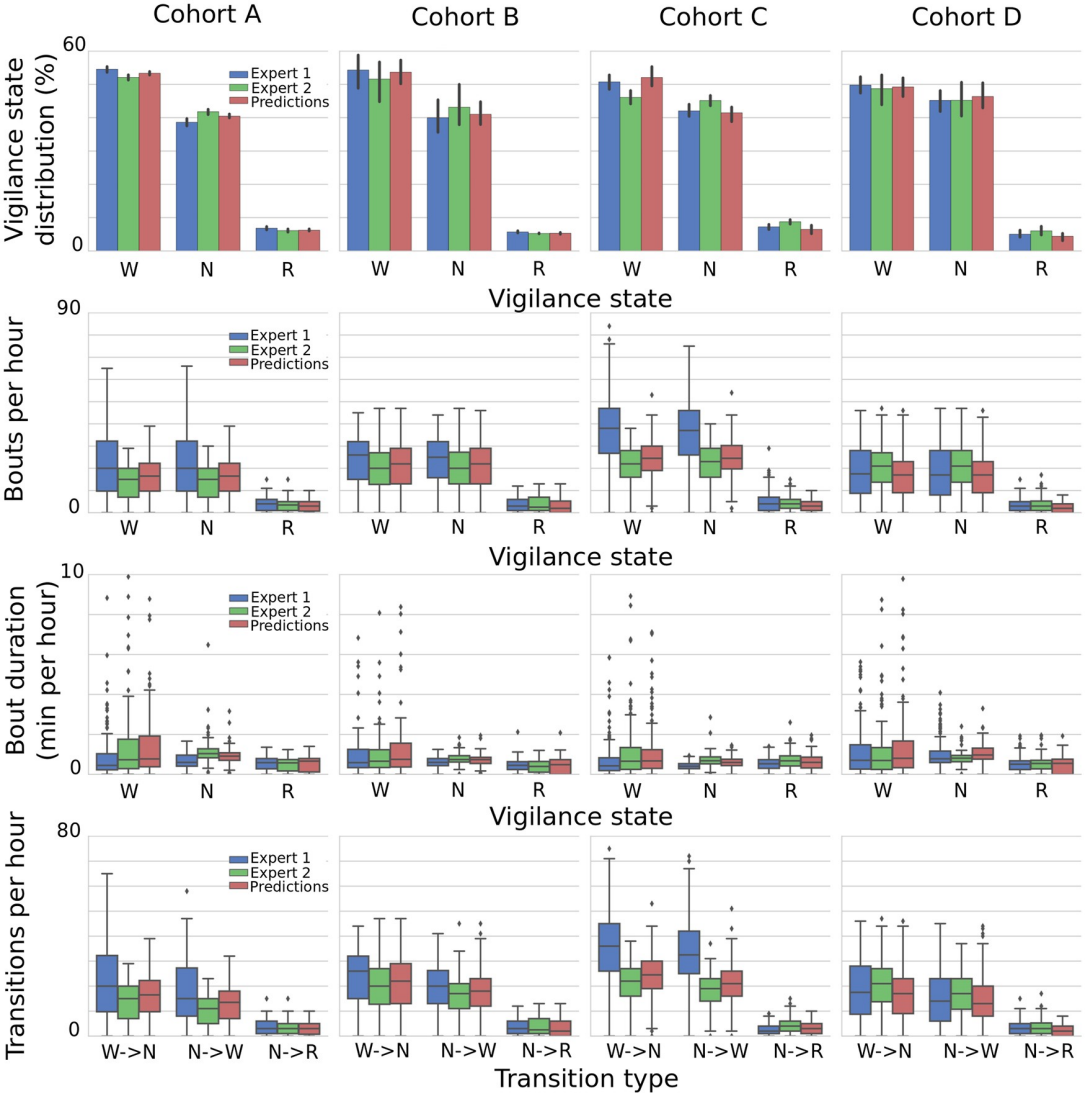
Predicting parameters of sleep architecture. Fraction of sleep (top row), bout duration (second row), number of bouts (third row), and number of sleep transitions (bottom row). Given are the box plots computed from the evaluation of these parameters per hour for each cohort, using data from each human scorer and from SPINDLE output. W→N, transition from WAKE to NREM; N→W, transition from NREM to WAKE; N→R, transitions from NREM to REM.

We computed and then compared the parameters of sleep architecture from all the three sources of vigilance state scorings, for each cohort separately. The goal was to evaluate the predictive power of SPINDLE in terms of several products of the output analysis: the fraction of vigilance states, bout duration, number of bouts and number of transitions. The resulting plots are given in Fig 9. Visually, it is clear that in most of the cases SPINDLE produced appealing predictions which represent a good balance between the two corresponding experts, thus potentially enabling less biased analysis. To statistically quantify the discrepancy between the predictions of SPINDLE and the human experts, we performed unpaired Student T-test between each pair of the corresponding distributions from Fig 9, comparing human experts and predictions. Statistical significance level was set at *p* < 0.05. Out of 48 independent output analysis shown in the figure, only in two cases the significance was reported between the predictions and both human experts, while the difference between two experts was non-significant: REM bout duration in cohort D (*p* = 0.016 and *p* = 0.004), and the number of REM bouts in the cohort C (both *p* < <0.01). In all other cases, our predictions were significantly close to at least one of the human experts. Furthermore, there were cases when the output of two human experts was significantly different, while SPINDLE’s predictions were not significantly different from any of the two: wake and NREM sleep fractions in the cohort A (*p* = 0.011 and *p* = 0.009), wake bout duration in the cohort C (*p* = 0.019), wake and NREM number of bouts in the cohort B (*p* = 0.011 and *p* = 0.032), and wake to NREM transition and vice versa in the cohort B (*p* = 0.013 and *p* = 0.009). These results clearly demonstrate that SPINDLE has great potential to improve the cross-expert/lab consistency in sleep classification.

#### Detecting mutation-induced differences between cohorts

**Fig 10:**
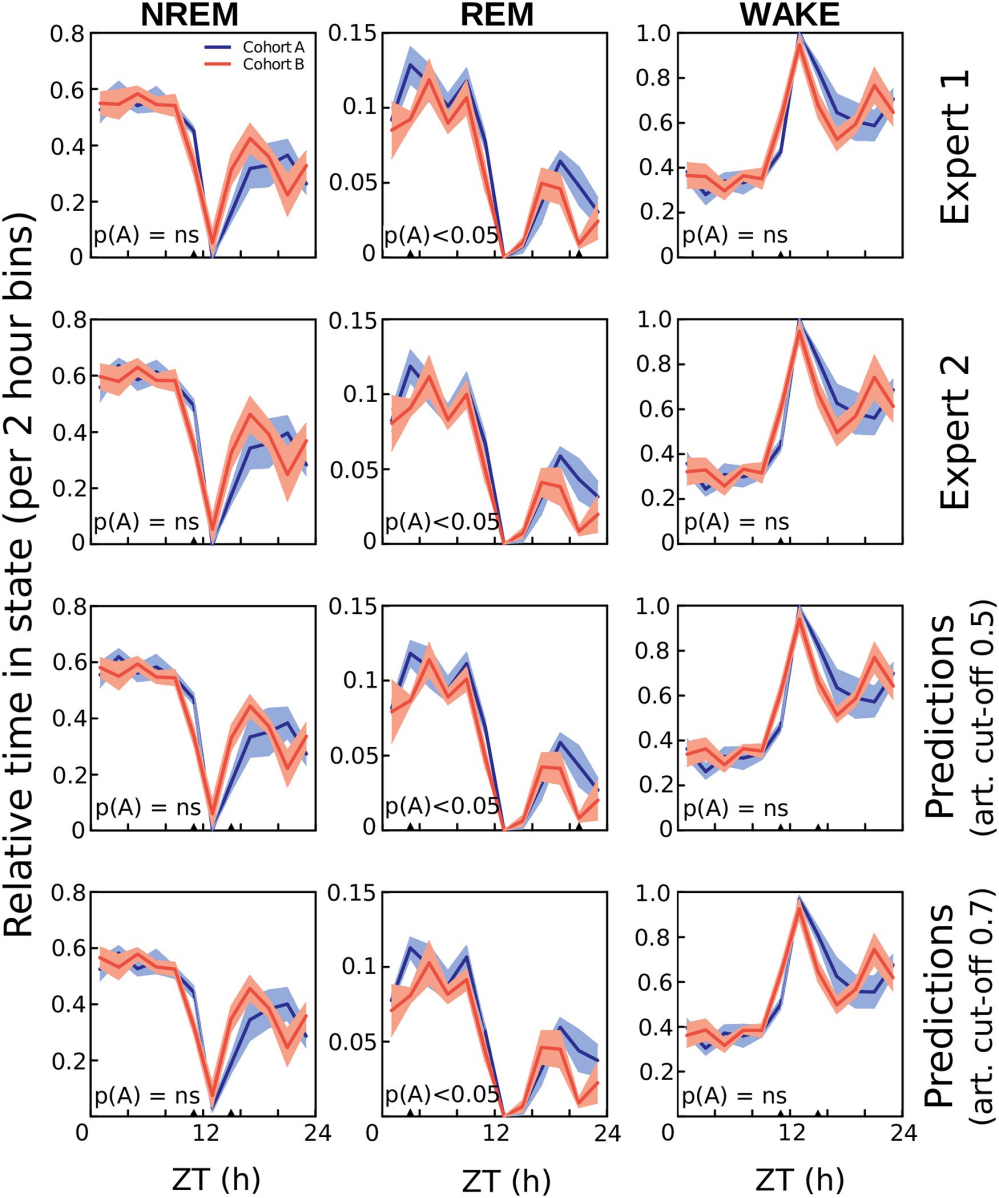
Detecting mutation-induced cohort differences in sleep timing. Fraction of NREM sleep, REM sleep and wakefulness per 2 hour intervals across 24 hours, in the cohorts A (wildtypes) and B (mutants). Results were evaluated from the scorings of the corresponding two experts and SPINDLE. The prediction curves were calculated for two different values of artifact threshold applied on the probabilistic output of the corresponding CNN (see step (d) in Fig 1). 0.5 is the default threshold, and indicates that only epochs with > 50% confidence of being non-corrupted were kept in the analysis. Similar procedure was applied for 0.7 artifact threshold. For measuring of the overall statistical significance, two-way ANOVA (marked as A) was used. ▲ *p* < 0.05 regions explain statistical differences of the corresponding 0.5Hz frequency bins measured using a two-tailed T-test with equal variances. The curves represent mean ± SEM. ZT, zeitgeber time.

**Fig 11:**
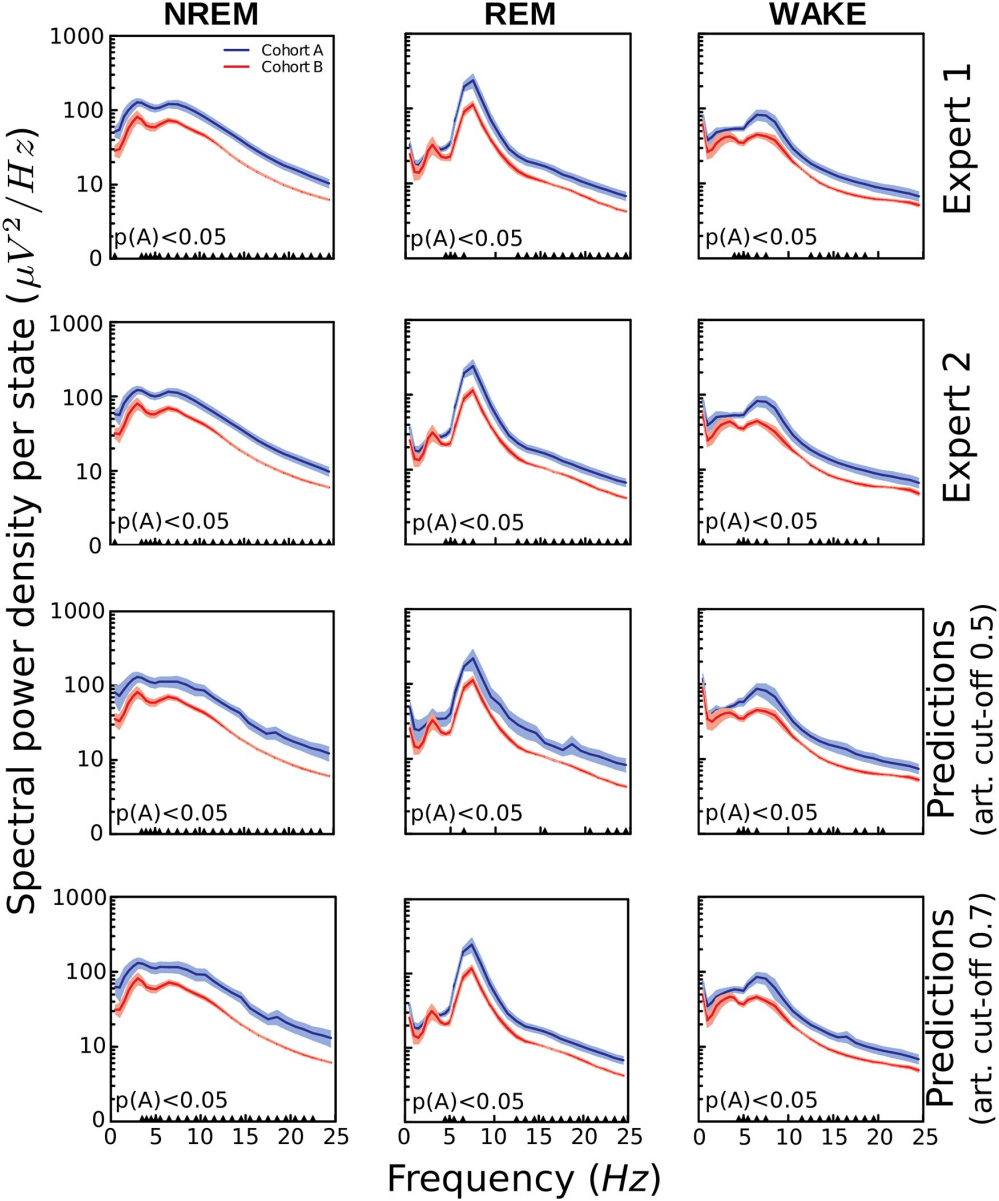
Detecting mutation-induced cohort differences in EEG spectra. EEG spectral power density plots of the cohort A (wildtypes) and B (mutants) during NREM, REM and wakefulness. Results were evaluated from the scorings of the corresponding two experts and SPINDLE. The prediction curves were calculated for two different values of artifact threshold applied on the probabilistic output of the corresponding CNN (see step (d) in Fig 1). 0.5 is the default threshold, and indicates that only epochs with > 50% confidence of being non-corrupted were kept in the analysis. Similar procedure was applied for 0.7 artifact threshold. For measuring of the overall statistical significance, two-way ANOVA (marked as A) was used. ▲ *p* < 0.05 regions explain statistical differences of the corresponding 0.5Hz frequency bins measured using a two-tailed T-test with equal variances. The curves represent mean ± SEM.

We investigated the capability of SPINDLE to detect significant differences in animal sleep patterns induced by experimental factors, thus emulating a real-life study. To that end, we post-processed the scorings of human experts and the predictions of SPINDLE to compare the cohorts A and B. The two cohorts were chosen because the corresponding animals had the same background, the only difference being a mutation of a gene in the mutant cohort B. We calculated the cohort differences with respect to sleep timing (Fig 10) and EEG power spectra (Fig 11). Both figures suggest that statistically significant discrepancies between cohorts were successfully identified by SPINDLE, when comparing to the ones identified by two human experts.

In addition, the figures illustrate the relevance of identifying artifactual data, especially when detecting significant statistical differences in EEG spectral power density cohort profiles. In particular, we performed equal output analysis for two different cut-off values applied on the probabilistic predictions generated by the CNN in step (d) in Fig 1. We showed that the strictness of artifact rejection criteria may have influence on designating the areas where the differences between compared cohorts appear. This is a relevant aspect to be considered when aiming for standardized and coherent systematic sleep studies. Since the artifacts are a major source of sleep scoring disagreement among human experts, it is often hard to find a common ground. However, the probabilistic nature of the SPINDLE framework could be useful in this regard, as it could offer some flexibility in adapting to the experiment-specific artifact rejection criteria.

### SPINDLE web service

Entire framework described in our study was integrated into a web service which can be found at https://sleeplearning.ethz.ch. This service ensures an easy access to the sleep researchers around the world and offers a possibility of accurate evaluation of EEG/EMG recordings in no time. Furthermore, we are continuously improving our framework aiming to create a self-sufficient environment for large scale animal analysis e.g. which would include output analysis in addition. Lastly, we would like to emphasize the two following aspects (i) the adaptable artifact threshold functionality; (ii) further technical considerations.

#### Adaptable artifact threshold

Due to the particularly high variations and subject-specific bias in designating epochs corrupted by artifacts, we decided to provide users with a certain flexibility in identifying artifactual epochs. To that end, SPINDLE offers an additional optional functionality which allows users to tune the artifact sensitivity by modifying the threshold from the output of the step (d) in Fig 1, therefore taking advantage of the probabilistic output of the corresponding CNN. A practical example of how this functionality may be utilized was mentioned earlier and is illustrated in Figs 10 and 11. Note that SPINDLE remains parameter-free, and that this flexibility is introduced to ensure a smoother road towards standardization with respect to the sensitive and not well defined rules for artifact identification.

#### Technical considerations

To ensure the successful utilization of the proposed framework we note down several technical aspects. First of all, even though our model was trained on a commonly used 2EEG/1EMG setup (employed in the generation of all the data presented here), we also enable users to use SPINDLE with 1EEG/1EMG experimental setting. We do that by multiplying the corresponding EEG channel. This ensures that the sleep labs with a different recording setup are still able to use SPINDLE. It is also worth noting, that data format must adhere precisely to the one described in detail at the above mentioned web platform.

#### Computation

The programming code underlying the sleep scoring server was written in torch (torch.ch) platform and will be made available through the server. To run the same instance of it locally no special requirements are necessary. We used the server framework for all our experiments. To speed up the learning and evaluation we used vectorized computation on a *GeForce GTX TITAN X* GPU card.

## Discussion

We described a new data processing architecture for fast, accurate and physiologically plausible automated sleep scoring of animal EEG/EMG recordings. SPINDLE is based on end-to-end learning and is capable of learning robust features which generalize across domains. From the robust benchmarking procedures that we employed we concluded that SPINDLE (i) showed de facto human level performance in the quality of sleep classification; (ii) significantly outperformed current state-of-the-art solutions; (iii) was able to preserve predictive performance across animals from different experimental settings, labs and species, without additional parameter calibration; (iv) was able to detect mutation-induced sleep alteration. Our extensive statistical evaluation suggests that the proposed method is in every way the equal of human experts, and holds great promise to improve cross-lab standardization of sleep analysis.

A part of SPINDLE’s success lies in the use of convolutional neural networks (CNNs). CNNs, a variant of deep neural networks, have enjoyed great success, particularly in the domain of natural signal processing [12, 15, 16]. They revolutionized the field of computer vision with their unprecedented success on image recognition tasks [12]. Here, we use them to combat a fundamental problem that has plagued automated sleep scoring: feature variance. Classical approaches based on manually crafted features are likely to fail when generalized across experiments simply because class-specific features or feature combinations may differ across data samples, or mutants, or species. Thus, because these models form their feature vectors from the energies of certain frequency bands, even small shifts of the spectral peaks may cause a different distribution of spectral energy with respect to the histogram bins, ruining predictive performance. All three methods that we tested alongside our own arguably suffer from this spectral pattern inconsistency issue related to manual feature extraction.

By contrast, a CNN extrapolates well across spectral profile variations much as a human expert would do. Furthermore, SPINDLE employs a hidden Markov model layer to describe probabilistic transitions between vigilance states. Thus, impossible transitions—for example wake to REM, known not to occur naturally—can be artificially suppressed by the user. Provided the assumptions about these physiological constraints hold, HMM generates physiologically plausible prediction sequences leading to improved performance.

Unlike other existing solutions which lack a principled way for distinguishing epochs corrupted by artifacts, SPINDLE employs an additional CNN to offer a dedicated computational tool for that task. The identification of artifacts from data is known to be quite challenging due to very large discrepancies in their annotation when different human experts are compared. Nevertheless, we showed that SPINDLE is performing well on this task as well, again reaching human expert level. Furthermore, to still provide some dose of flexibility in this highly biased and human expert-dependent procedure, our framework offers additional functionality which allows users to adapt the degree of artifact rejection. Our framework remains parameter-free and this optional functionality exist to ensure smoother convergence towards full standardization of the sleep scoring procedure.

There are some relevant design decisions worth of noting. Firstly, in our work we used *out-of-the box CNN*, and not any of the commonly used architectures such as AlexNet [12] or ResNet [28]. We empirically found that increasing the depth and width of our network did not led to any improvements, and hence found no reason to e.g. add additional layers to the main architecture. Our hypothesis is that the structural complexity of “spectrograms” is not comparable to the structural complexity of natural images, thus there were additional benefits in having an increased complexity in our CNN architecture. Secondly, in contrast to some related work in the domain of human sleep scoring [8, 19–21, 24], our final architecture does not include memory models i.e. recurrent neural networks (RNNs). We found that their inclusion did not lead to any performance improvements and moreover was harmful to across-domain generalization. Our tests indicated that RNN tends to overfit to subject-specific dynamics and hence makes it difficult to extrapolate knowledge across environments. On the other hand, even though not mutually exclusive, HMMs offer more fine-grained control over the transition dynamics. Secondly, we utilized separate CNNs for artifacts and vigilance states for the following reasons: (i) semantically, the patterns in data are different—vigilance states are recognized by somewhat predefined rules while the artifacts are data outliers; (ii) the described setup allows us to identify three different types of artifacts, thus more faithfully emulating visual scoring procedure; (iii) we found that the proposed framework leads to better predictive performance.

Finally, as a part of our future work, we intend to investigate the capabilities of and re-fine our method further in subsequent studies possibly involving other experimental paradigms such as sleep deprivation for example, which might possibly cause other types of distortions of spectral profiles. Another interesting aspect would involve including more human scorers and consequently applying more principled ways of combining their knowledge (e.g. see [29]).

## Materials and methods

### Experimental data acquisition

We detail the steps in collecting the data previously summarized in Table 1. In total, 4 animal cohorts containing 22 animals were acquired from 3 independent sleep labs. Animal studies were performed by authorized researchers according to all applicable laws and regulations of the cantons of Zürich (BrownLab, BaumannLab) and Bern (TidisLab), and were each approved by the relevant cantonal authorities. Each sleep recording consists of a pair of EEG signals and an EMG signal simultaneously recorded. Manual labeling of wake-sleep states based on EEG/EMG signals was performed by trained experts from the corresponding sleep labs on all consecutive 4 second epochs. Raw EEG traces were visually inspected offline and scored in three vigilance states, wakefulness, NREM sleep and REM sleep. Wakefulness was defined based on increased EMG activity for more than 50% of the epoch duration. NREM sleep was defined by reduced EMG activity and increased EEG power in < 4Hz frequency ranges. REM sleep was characterized based on low EEG power in > 4Hz oscillations and high EEG power in 6-9Hz frequency bands and intermediate muscle tone. Unclear stages or technical artifacts were excluded and subsequently labeled as artifacts.

#### BrownLab data set

It contains sleep recordings of two cohorts: the cohort A containing 4 wildtype mice and the cohort B containing 4 mice with a genetic mutation. All mice were kept in Macrolon cages (36x20x35cm) with food and water ad libitum, maintained at a 12 hour light-dark cycle (light onset 07.00 AM) in normal cages prior to surgery, and then in open-top cages with counterbalanced swivel-attached cables during and between sleep recordings. For the EEG recordings, mice were implanted epidurally with gold-plated miniature screws (0.9mm diameter) under constant isofluran inhalation anesthesia. Analgesia was given i.p. at 0.1mg/kg during the surgery. The coordinates of EEG electrodes were as follows; frontal derivation was placed 1mm anterior to bregma, 1.5mm lateral to mid-line, the parietal derivation was placed 3mm posterior to bregma and 2mm lateral to mid-line. The reference was placed over the cerebellum (2mm posterior to lambda on the midline). Two gold wires (0.2mm diameter) were inserted bilaterally in the neck muscle for EMG recordings. The screws were connected to stainless steel wires and fixed on the skull with acrylic dental concrete. The mice were tethered to a swivel throughout the entire experiment and were allowed to recover for 4 to 7 days before any further handling. The EEG and EMG signals were amplified (amplification factor, 2000), filtered (highpass filter: –3 dB at 0.016 Hz; low-pass filter: –3 dB at 40 Hz) sampled with 512 Hz, digitally filtered [EEG: low-pass finite impulse response (FIR) filter, 25 Hz; EMG: bandpass FIR filter, 20–50 Hz or 10-30Hz], and stored with a resolution of 128 Hz. Before each recording, the EEG and EMG channels were calibrated with a 10 Hz, 300 μV peak-to-peak sine wave.

#### BaumannLab data set

It contains sleep recordings of the cohort C which consists of 8 rats. The rats were implanted with EEG/EMG electrodes for recording of vigilance states using a protocol slightly modified from [30]. Briefly, four stainless steel miniature screws (Hasler, Switzerland), one pair for each hemisphere, were inserted bilaterally into the rats’ skull following specific stereotactic coordinates: the anterior electrodes were implanted 3mm posterior to bregma and 2mm lateral to the midline, and the posterior electrodes 6mm posterior to bregma and 2mm lateral to the midline. For monitoring of muscle tone, a pair of gold wires served as EMG electrodes and was inserted into the rats’ neck muscle. All electrodes were connected to stainless steel wires, further connected to a head piece (Farnell, #M80-8540842, Switzerland) and fixed to the skull with dental cement. Bilateral 24 hour EEG/EMG signals were recorded in freely-moving rats. For this purpose, the animals were transferred to special recording cages with food and water available ad libitum, where they had an adaptation period of two days before recordings took place. EEG and EMG were sampled at 200 Hz, signals were amplified, filtered and converted into analog-to-digital signals. Hardware EMBLA and Somnologica-3 software (Medcare Flaga, Iceland) were used. Activity in the 50-Hz band was discarded from the analysis because of power line artifacts.

#### TidisLab data set

It contains sleep recordings of the cohort D.*C57Bl6 mice* were used, 11-14 weeks of age, from Charles Rivers Laboratories, Germany. Animals were housed in individual custom-designed polycarbonate cages at constant temperature (22 ± 1 °C), humidity (30–40%), and circadian cycle (12 hour light-dark cycle, lights on at 08:00). Food and water were available ad libitum. Animals were treated according to protocols and guidelines approved by the Veterinary office of the Canton of Bern, Switzerland (License number BE 113/13). Animals were housed in IVC cages in groups of 2–5 before instrumentation. After implantation, all mice were housed individually. Animals were habituated to the recording cable in their open-top home cages (300 × 170 mm). Animals were anaesthetized in isoflurane in oxygen and mounted in a stereotaxic frame. Saline 10ml/kg and meloxicam 5mg/kg were given subcutaneously. The skin on the head was shaved and aseptically prepared, and lidocaine 2mg/kg infused subcutaneously at the incision site. A single longitudinal midline incision was made from the level of the lateral canthus of the eyes to the lambda skull suture. Two stainless steel screws were placed in the skull over the parietal cortex to measure EEGs and two bare-ended wires sutured to the trapezius muscle of the neck to record EMG. The implant was stabilized using a methyl methacrylate cement and the animal allowed to recover in the home cage on top of a heating mat. Animals were allowed a minimum of 5 days to recover before starting recordings. Habituation to the cables was performed up to 8 hours per day until the animals had nested and resumed a normal sleep-wake cycle. For recordings, mice were connected to the AM system and data sampled at 512 Hz. For each mouse, 24 hour baseline sleep was recorded, while animal was allowed to move freely in the cage. Recorded EEG/EMG signals were down-sampled to 128 Hz, after applying a low pass filter (Chebyshev Type I, order 8, low pass edge frequency of 50 Hz, passband ripple of 0.05 dB) to prevent aliasing.

### Data preprocessing

**Fig 12:**
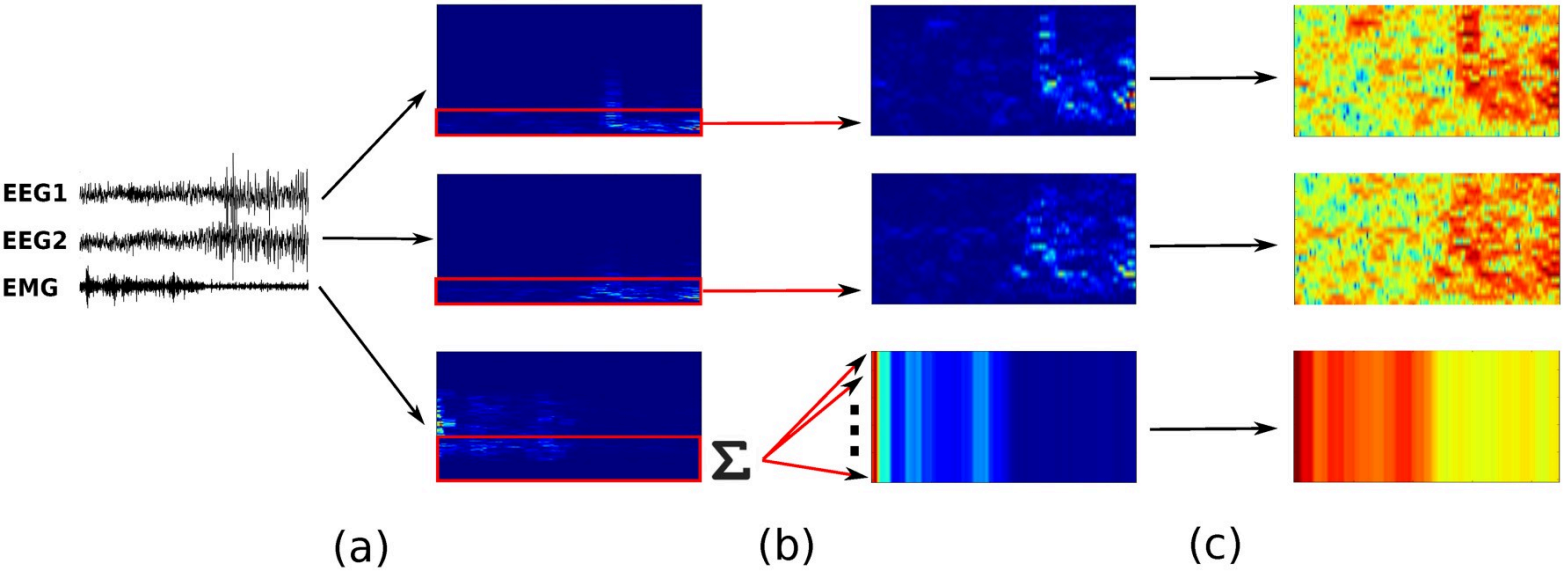
Data preprocessing and CNN input preparation. The figure depicts the creation of the three-channel two-dimensional input for the CNNs. In step (a) raw time series of EEG/EMG signals are separately transformed into the corresponding time-frequency domain (power spectrum density is computed) via a sequence of short Fourier transforms applied to overlapping Hamming windows. In step (b) EEG signals are band-pass filtered (0.5–24*Hz*) and EMG power is integrated over frequency range (0.5-30Hz) resulting in one-dimensional representation of muscle activity change over time. Furthermore, one-dimensional representation of EMG is converted into the two-dimensional one by a multiplication of the signal. Finally, in step (c) the data is log transformed and standardized per frequency component.

The data preprocessing module (step (c) in Fig 1) serves primarily to form the input for the convolutional neural networks (CNNs). We subject EEG/EMG signals to a sequence of transformations which enhance the learning process and consequently improve classification performance. The transformations are performed per animal and are meant to diminish non-informative differences in subject specific spectral patterns. The preprocessing procedure is illustrated in Fig 12. Both EEG signals are first resampled to the frequency of 128Hz to neutralize the differences in sampling rates coming from different recording devices. Resampled EEGs are then transformed into the time-frequency domain by applying fast Fourier transform on overlapping frames of size 256 (corresponds to 2 seconds) with steps of size 16. Hamming windows were applied to reduce edge effects. Power spectral density (PSD) in time-frequency representation is estimated as squared magnitude of the Fourier transform. Each of the two dimensional spectrograms constructed from EEG signals is treated as a separate feature map on top of which the CNNs convolve. This is analogous to the well-known CNN image classification architectures where the input consists of 3 RGB channels [12]. EEG spectrograms are additionally band-pass filtered (0.5-24Hz), as we experimentally determined that classification performance remains unaffected. Both time-frequency channels are then transformed to log scale and finally each channel is per frequency component standardized (zero mean / unit variance).

The EMG signal on the other hand carries the information about muscle activity of evaluated subjects. The total energy of EMG indicates the activity of the corresponding muscle. To decrease the noise we compute signal energy by integrating PSD over a limited frequency band (0.5-30Hz). In other words, we sum up the rows in our time-frequency representation within the given frequency range (see Fig 12). This leaves us with one-dimensional signal which measures the change in muscle activity over time. However, in order to form a consistent input for CNN with respect to two-dimensional representations of EEG, we introduce an additional dimension by repeating the signal as illustrated in the figure. This way of forming input is beneficial because in each time instance the CNN filters can relate the total EMG signal power with spectral patterns in different regions of the frequency axis.

### CNN architecture and training

A Convolutional Neural Networks (CNN) [12, 31] is an artificial neural network most commonly composed of a sequence of (i) convolutional layers which learn high level signal representations; (ii) pooling layers which increase the translational feature invariance; (iii) dense layers which learn high-level feature combinations in a discriminative manner; and (iv) a softmax layer which generates class probabilities. Details of these layers follow below. For a more thorough introduction to CNN we refer the reader to [32].

#### Convolutional layer

The convolutional layer implements a filter operation *h* of size *m* × *m*. The resulting value at some neuron *x*_*ij*_ in layer *ℓ* can be written as:

$$\begin{array}{c} x_{ij}^{\ell }=\sum_{a=0}^{m-1}\sum_{b=0}^{m-1}h_{ab}y_{\left(i+a)(j+b\right)}^{\ell -1}+\beta_{ij}^{\ell } \end{array}$$ (3)

where *h*_*ab*_ is the weight of the filter in the point (*a*, *b*) of the *m* × *m* grid, $y_{**}^{\ell -1}$ are the corresponding outputs of the previous layer and $\beta_{ij}^{\ell }$ is the bias term. After the filter, a non-linear function *σ* is applied to all the neurons in layer *ℓ* independently: $y_{ij}^{\ell }=\sigma \left(x_{ij}^{\ell }\right)$. We used the well-established rectifier linear unit (ReLU) [33]: $\sigma \left(x_{ij}^{\ell }\right)=\max\left(0,x_{ij}^{\ell }\right)$.

#### Max-pooling layer

**Fig 13:**
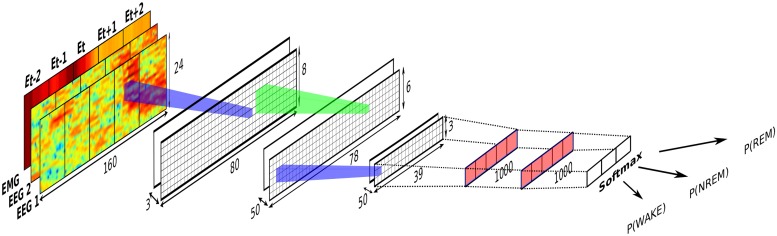
Sleep scoring CNN architecture. Presented are the architectural details of the CNN which estimates the probability distribution over vigilance states for the target E_*t*_. Input to the CNN is formed as shown in Fig 12. The CNN operates over four neighboring epochs E_*t*−2_, E_*t*−1_, E_*t*+1_ and E_*t*+2_ to capture the contextual information. Illustrated CNN consists of two max-pooling layers (depicted in blue), one convolutional layer (depicted in green), and two fully-connected layers (depicted in red). At the very end, a softmax layer outputs class probabilities. The dimensions of the first max-pooling layer are (width, height) = (2, 3) with the corresponding strides (2, 3). The dimensions of the second max-pooling layer are (2, 2) with the corresponding strides (2, 2). The dimensions of the convolutional kernel are (3, 3) with the corresponding strides (1, 1).

The max-pooling layer down-samples the previous layer by choosing only the maximal value of non-overlapping rectangles of size *m*_*h*_ × *m*_*w*_, see the blue cones in Fig 13 for an illustration. The max-pooling layer provides translation-invariance and reduces the number of parameters of the CNN.

#### Dense layer

The last layer in a CNN before the softmax activation function is usually a fully-connected dense layer that connects all the neurons in the previous layer. The resulting value at some neuron $x_{i}^{\ell }$ in the layer *ℓ* can be written as:

$$\begin{array}{c} x_{i}^{\ell }=\sum_{j=0}^{n}\alpha_{ij}\cdot y_{j}^{\ell -1}+\beta_{i}^{\ell } \end{array}$$ (4)

where *α*_*ij*_ is the weight corresponding to output $y_{j}^{\ell -1}$ of the previous layer, and $\beta_{i}^{\ell }$ is the bias term.

#### Softmax layer

The last layer is a softmax layer, given by Eq 5 where *C* is one of *K* classes and *y*^(*C*)^ the output of the previous layer with respect to the class *C*. It is used to get normalized class probabilities:

$$\begin{array}{c} P\left(C\right)=\frac{e^{y^{\left(C\right)}}}{\sum_{i=1}^{K}e^{y^{\left(C_{i}\right)}}} \end{array}$$ (5)

In our work we adopted this kind of convolutional neural network architecture. Discriminative features are learned in an end-to-end fashion from three-channel time-frequency signals (the output in Fig 12). Apart from the number of output units, design-wise, two CNNs in our framework are equivalent, thus here we refer only to the CNN used for the classification of vigilance states (step (e) in Fig 1). The architectural details are given in Fig 13. To encapsulate the contextual information the CNN convolves over the target epoch, but also over the surrounding neighbor epochs, two from each side. The variability of spectral profiles (previously discussed in Results) is naturally solved through the discriminative learning of translation invariant features. Namely, by convolving and max-pooling over the frequency domain, the CNN becomes agnostic to small shifts in spectral energy distribution which appear when comparing different animals. On the other hand, by operating over time domain we become agnostic to where the spectral patterns appear within the evaluated region of the input signal.

Weight learning was performed via back-propagation using the optimization algorithm called Adam [34]. The weight decay rates of first and second moment were set to 0.9 and 0.99 as suggested in the original paper. Prior to back-propagation, weights were randomly initialized as described in [35]. To account for the class imbalance (e.g. see the top row in Fig 9) the Adam optimizer was governed by a class-weighted cross-entropy loss function. Given the observation *x* and the true class *C* ∈ {*C*_1_ = *WAKE*, *C*_2_ = *NREM*, *C*_3_ = *REM*} the corresponding loss is calculated as:

$$\begin{array}{c} \text{loss}\left(C,x\right)=-w\left(C\right)\cdot \log\left(\frac{e^{f(C,x)}}{\sum_{i=1}^{3}e^{f(C_{i},x)}}\right)=-w\left(C\right)\cdot \left(f\left(C,x\right)-\log\left(\sum_{i=1}^{3}e^{f(C_{i},x)}\right)\right) \end{array}$$ (6)

where *f*(*C*, *x*) is the output of the last fully-connected layer corresponding to class *C* and *w*(*C*) is the weight of that class. Class weights *w*(*C*_*i*_) were for each mini-batch independently set with respect to the class sample ratio within that mini-batch. Final gradient was computed as the normalized sum of individual losses within the mini-batch:

$$\begin{array}{c} \text{batch}\_\text{loss}\left(C^{1..M},x^{1..M}\right)=\frac{1}{M}\sum_{i=1}^{M}\text{loss}\left(C^{i},x^{i}\right) \end{array}$$ (7)

Learning rate was set to 5 ⋅ 10^−5^ and each mini-batch contained M = 100 samples. To further regularize the learning procedure we applied the dropout with the probability of 50% to the fully connected layers and allocated 10% of the data from the training set for early stopping. Overall, our CNN contained 6.8M parameters in total, and converged on the held-out set (10%) already after 5 full iterations over the training data.

### Constraining transition dynamics with HMM

To obtain a finer-grained modeling control over the dynamics of vigilance state transitions we utilize a hidden Markov model (HMM). Broadly speaking, HMMs are tools for representing probability distributions over sequences of indirectly observable states. A first-order HMMs is fully specified by the probability distribution of the initial state *P*(*S*_1_), the matrix of transition probabilities between neighboring states *P*(*S*_*t*_|*S*_*t*−1_) and the output model defined by the emission likelihoods *P*(*Y*_*t*_|*S*_*t*_) where *Y*_*t*_ is the indirect observation of variable *S*_*t*_. In the context of our problem, the hidden state in some moment *t* is the vigilance state of the brain *S*_*t*_ ∈ {*WAKE*, *REM*, *NREM*}, while the observation *Y*_*t*_ is the corresponding region of EEG/EMG signal (in Fig 13 that would be *epoch*_*t*−2_ to *epoch*_*t*+2_) from which hypothetically the state *S*_*t*_ can be inferred. Our goal in the training time is to find the optimal parameters λ = [*P*(*Y*_*t*_|*S*_*t*_), *P*(*S*_*t*_|*S*_*t*−1_), *P*(*S*_1_)] of the HMM, and then to use learned parameters later in the test time to obtain the most probable sequence of vigilance states given input signal: $\underset{S_{1..N}}{\text{argmax}}\;P\left(S_{1..N}|\lambda ,Y_{1..N}=\text{EEG/EMG}\right)$ where N is the number of epochs.

Firstly, to find the indirect observation emission likelihoods *P*(*Y*_*t*_|*S*_*t*_) we use the probabilities generated by the discriminatively trained CNN and we treat them as the model output. Namely, CNN produces posterior probabilities over states *P*(*S*_*t*_|*Y*_*t*_) which can be used for computing HMM emission likelihoods *P*(*Y*_*t*_|*S*_*t*_). They are connected by the following equation which is derived directly from the Bayes rule:

$$\begin{array}{c} \frac{P(Y_{t}|S_{t})}{P(Y_{t})}=\frac{P(S_{t}|Y_{t})}{P(S_{t})} \end{array}$$ (8)

**Fig 14:**
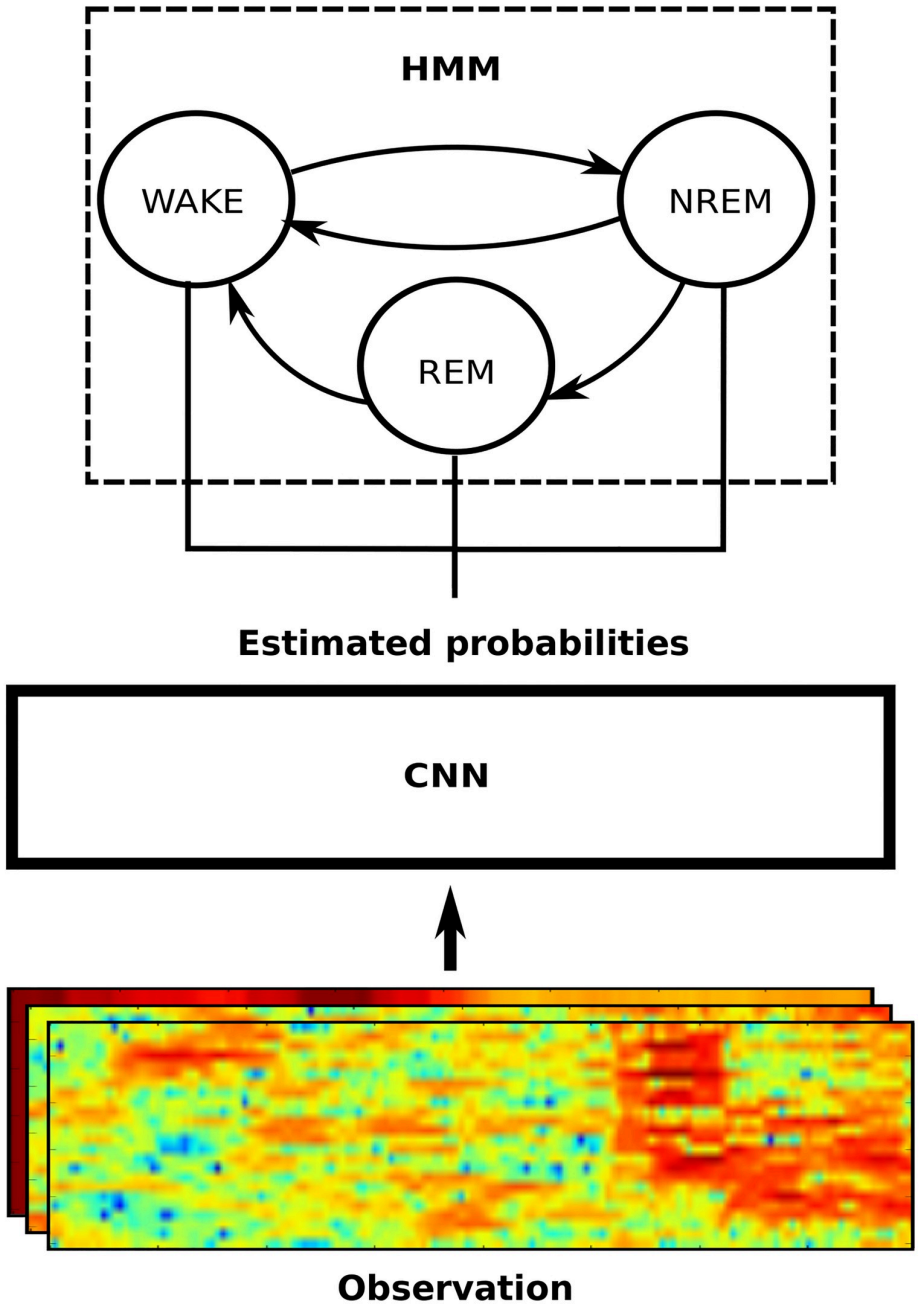
CNN-HMM for constraining state transitions. The figure illustrates how the HMM is used on top of the CNN to enforce prediction sequences which adhere to physiological constraints. In particular, we disallow *REM* → *NREM* and *WAKE* → *REM* vigilance state transitions. The constraints are encoded through the transition probability matrix of the HMM, and the observation likelihoods are implicitly calculated by the CNN.

Secondly, without loss of generality and any substantial effect to the predictive performance we may assume that all initial states *P*(*S*_1_) are equal. Finally, the last component of the HMM is the probability transition matrix *P*(*S*_*t*_|*S*_*t*−1_) which is of size 3 × 3 in our case. In order to generate physiologically feasible prediction sequences, when applicable, using the probability transition matrix we can embed the domain knowledge on infeasible vigilance state transitions. An illustration is given in Fig 14. Whenever it is known in advance that certain transitions are not valid such as *REM* → *NREM* and *WAKE* → *REM* [36, 37] which is the case for all the recordings in our data set, we can zero out the corresponding entries in the probability transition matrix. Since there is an additional constraint that the rows of the transition matrix must sum up to 1, this leaves us with effectively only four remaining free parameters. These can be set to be of equal value, or tuned additionally to improve the smoothing of the vigilance state sequence estimates. Having specified the model, new posterior probabilities over vigilance states are generated as follows:

$$\begin{array}{c} \begin{array}{c} P\left(S_{t}|Y_{t},S_{t-1}\right)=\frac{P\left(Y_{t}|S_{t}\right)P\left(S_{t}|S_{t-1}\right)}{P(Y_{t})}=\frac{P\left(S_{t}|Y_{t}\right)P\left(S_{t}|S_{t-1}\right)}{P(S_{t})} \end{array} \end{array}$$ (9)

where we used Eq 8 to obtain the last equality. Since the CNN is trained in a class-balanced way which compensates for an unequal class ratio in the training set, the prior probabilities of states *P*(*S*_*t*_) may be assumed to be equal. Finally, using the specified HMM model we may now simply apply Viterbi decoding to find the most probable sequence of vigilance states (the output of step (f) in Fig 1).

In summary, the HMM allows us to combine the CNN estimates used for computing emission likelihoods *P*(*Y*_*t*_|*S*_*t*_) (*observation modeling*) with the knowledge encoding capability of the HMM probability transition matrix *P*(*S*_*t*_|*S*_*t*−1_) (*dynamics modeling*) to produce more plausible and consequently more accurate prediction sequences. It is also worth noting that the commonly advocated problem of HMMs that the observations are assumed to be independent (given state) is treated through the inclusion of neighboring epochs into the convolving field of the CNN (recall Fig 13).

## References

[1] Mignot E. Why we sleep: the temporal organization of recovery. PLoS biology. 2008 4 29;6(4):e106 10.1371/journal.pbio.0060106 18447584 PMC2689703

[2] Vorster AP, Krishnan HC, Cirelli C, Lyons LC. Characterization of sleep in Aplysia californica. Sleep. 2014 9 1;37(9):1453–63. 10.5665/sleep.3992 25142567 PMC4153062

[3] Borbély AA, Daan S, Wirz-Justice A, Deboer T. The two-process model of sleep regulation: a reappraisal. Journal of sleep research. 2016 4 1;25(2):131–43. 10.1111/jsr.12371 26762182

[4] Rempe MJ, Clegern WC, Wisor JP. An automated sleep-state classification algorithm for quantifying sleep timing and sleep-dependent dynamics of electroencephalographic and cerebral metabolic parameters. Nature and science of sleep. 2015;7:85 10.2147/NSS.S84548 26366107 PMC4562753

[5] Kohtoh S, Taguchi Y, Matsumoto N, Wada M, HUANG ZL, Urade Y. Algorithm for sleep scoring in experimental animals based on fast Fourier transform power spectrum analysis of the electroencephalogram. Sleep and Biological Rhythms. 2008 7 1;6(3):163–71. 10.1111/j.1479-8425.2008.00355.x

[6] Bastianini S, Berteotti C, Gabrielli A, Del Vecchio F, Amici R, Alexandre C, Scammell TE, Gazea M, Kimura M, Martire VL, Silvani A. SCOPRISM: a new algorithm for automatic sleep scoring in mice. Journal of neuroscience methods. 2014 9 30;235:277–84. 10.1016/j.jneumeth.2014.07.018 25092499

[7] Yaghouby F, O’Hara BF, Sunderam S. Unsupervised Estimation of Mouse Sleep Scores and Dynamics Using a Graphical Model of Electrophysiological Measurements. International journal of neural systems. 2016 6;26(04):1650017 10.1142/S0129065716500179 27121993

[8] Dong H, Supratak A, Pan W, Wu C, Matthews PM, Guo Y. Mixed neural network approach for temporal sleep stage classification. IEEE Transactions on Neural Systems and Rehabilitation Engineering. 2018 2;26(2):324–33. 10.1109/TNSRE.2017.2733220 28767373

[9] Rytkönen KM, Zitting J, Porkka-Heiskanen T. Automated sleep scoring in rats and mice using the naive Bayes classifier. Journal of neuroscience methods. 2011 10 30;202(1):60–4. 10.1016/j.jneumeth.2011.08.023 21884727

[10] Sunagawa GA, Séi H, Shimba S, Urade Y, Ueda HR. FASTER: an unsupervised fully automated sleep staging method for mice. Genes to Cells. 2013 6 1;18(6):502–18. 10.1111/gtc.12053 23621645 PMC3712478

[11] Hinton G, Deng L, Yu D, Dahl GE, Mohamed AR, Jaitly N, Senior A, Vanhoucke V, Nguyen P, Sainath TN, Kingsbury B. Deep neural networks for acoustic modeling in speech recognition: The shared views of four research groups. IEEE Signal Processing Magazine. 2012 11;29(6):82–97. 10.1109/MSP.2012.2205597

[12] Krizhevsky A, Sutskever I, Hinton GE. Imagenet classification with deep convolutional neural networks. InAdvances in neural information processing systems 2012 (pp. 1097-1105).

[13] Karpathy A, Toderici G, Shetty S, Leung T, Sukthankar R, Fei-Fei L. Large-scale video classification with convolutional neural networks. InProceedings of the IEEE conference on Computer Vision and Pattern Recognition 2014 (pp. 1725-1732).

[14] Donahue J, Anne Hendricks L, Guadarrama S, Rohrbach M, Venugopalan S, Saenko K, Darrell T. Long-term recurrent convolutional networks for visual recognition and description. InProceedings of the IEEE conference on computer vision and pattern recognition 2015 (pp. 2625-2634). 10.1109/TPAMI.2016.2599174 27608449

[15] Kim Y. Convolutional neural networks for sentence classification. arXiv preprint arXiv:1408.5882. 2014 Aug 25.

[16] Graves A, Mohamed AR, Hinton G. Speech recognition with deep recurrent neural networks. InAcoustics, speech and signal processing (icassp), 2013 ieee international conference on 2013 May 26 (pp. 6645-6649). IEEE.

[17] Yu, Dong, and Li Deng. AUTOMATIC SPEECH RECOGNITION. SPRINGER LONDON Limited, 2016.

[18] Chan W, Jaitly N, Le Q, Vinyals O. Listen, attend and spell: A neural network for large vocabulary conversational speech recognition. InAcoustics, Speech and Signal Processing (ICASSP), 2016 IEEE International Conference on 2016 Mar 20 (pp. 4960-4964). IEEE.

[19] Hsu YL, Yang YT, Wang JS, Hsu CY. Automatic sleep stage recurrent neural classifier using energy features of EEG signals. Neurocomputing. 2013 3 15;104:105–14. 10.1016/j.neucom.2012.11.003

[20] Längkvist M, Karlsson L, Loutfi A. Sleep stage classification using unsupervised feature learning. Advances in Artificial Neural Systems. 2012 7 24;2012.

[21] Supratak A, Dong H, Wu C, Guo Y. DeepSleepNet: A model for automatic sleep stage scoring based on raw single-channel EEG. IEEE Transactions on Neural Systems and Rehabilitation Engineering. 2017 11;25(11):1998–2008. 10.1109/TNSRE.2017.2721116 28678710

[22] Sors A, Bonnet S, Mirek S, Vercueil L, Payen JF. A convolutional neural network for sleep stage scoring from raw single-channel EEG. Biomedical Signal Processing and Control. 2018 4 30;42:107–14. 10.1016/j.bspc.2017.12.001

[23] Bashivan P, Rish I, Yeasin M, Codella N. Learning representations from EEG with deep recurrent-convolutional neural networks. arXiv preprint arXiv:1511.06448. 2015 Nov 19.

[24] Zhao M, Yue S, Katabi D, Jaakkola TS, Bianchi MT. Learning sleep stages from radio signals: A conditional adversarial architecture. InInternational Conference on Machine Learning 2017 Jul 17 (pp. 4100-4109).

[25] Franken P, Malafosse A, Tafti M. Genetic variation in EEG activity during sleep in inbred mice. American Journal of Physiology-Regulatory, Integrative and Comparative Physiology. 1998 10 1;275(4):R1127–37. 10.1152/ajpregu.1998.275.4.R1127 9756543

[26] Abdel-Hamid O, Mohamed AR, Jiang H, Deng L, Penn G, Yu D. Convolutional neural networks for speech recognition. IEEE/ACM Transactions on audio, speech, and language processing. 2014 10;22(10):1533–45. 10.1109/TASLP.2014.2339736

[27] Tafti M, Petit B, Chollet D, Neidhart E, De Bilbao F, Kiss JZ, Wood PA, Franken P. Deficiency in short-chain fatty acid *β*-oxidation affects theta oscillations during sleep. Nature genetics. 2003 7;34(3):320 10.1038/ng1174 12796782

[28] He K, Zhang X, Ren S, Sun J. Deep residual learning for image recognition. InProceedings of the IEEE conference on computer vision and pattern recognition 2016 (pp. 770-778).

[29] ANSARI Amir Hossein, et al Weighted Performance Metrics for Automatic Neonatal Seizure Detection Using Multiscored EEG Data. IEEE journal of biomedical and health informatics, 2018, 22 Jg., Nr. 4, S. 1114–1123. 10.1109/JBHI.2017.2750769 28910781

[30] Baumann CR, Kilic E, Petit B, Werth E, Hermann DM, Tafti M, Bassetti CL. Sleep EEG changes after middle cerebral artery infarcts in mice: different effects of striatal and cortical lesions. Sleep. 2006 10 1;29(10):1339–44. 10.1093/sleep/29.10.1339 17068988

[31] LeCun Y, Bengio Y. Convolutional networks for images, speech, and time series. The handbook of brain theory and neural networks. 1995 4;3361(10):1995.

[32] Goodfellow I, Bengio Y, Courville A, Bengio Y. Deep learning. Cambridge: MIT press; 2016 11 18.

[33] Nair V, Hinton GE. Rectified linear units improve restricted boltzmann machines. InProceedings of the 27th international conference on machine learning (ICML-10) 2010 (pp. 807-814).

[34] Kingma DP, Ba J. dam: A method for stochastic optimization. ICLR, 2015.

[35] LeCun Y, Bottou L, Orr GB, Müller KR. Efficient backprop. InNeural networks: Tricks of the trade 1998 (pp. 9-50). Springer, Berlin, Heidelberg.

[36] Benington JH, Heller HC. REM-sleep timing is controlled homeostatically by accumulation of REM-sleep propensity in non-REM sleep. American Journal of Physiology-Regulatory, Integrative and Comparative Physiology. 1994 6 1;266(6):R1992–2000. 10.1152/ajpregu.1994.266.6.R1992 8024056

[37] Borb AA, Achermann P. Sleep homeostasis and models of sleep regulation. Journal of biological rhythms. 1999 12;14(6):559–70. 10.1177/074873099129000894 10643753

